# More Than the Sum of Its Parts: Disrupted Core Periphery of Multiplex Brain Networks in Multiple Sclerosis

**DOI:** 10.1002/hbm.70107

**Published:** 2024-12-30

**Authors:** Giuseppe Pontillo, Ferran Prados, Alle Meije Wink, Baris Kanber, Alvino Bisecco, Tommy A. A. Broeders, Arturo Brunetti, Alessandro Cagol, Massimiliano Calabrese, Marco Castellaro, Sirio Cocozza, Elisa Colato, Sara Collorone, Rosa Cortese, Nicola De Stefano, Linda Douw, Christian Enzinger, Massimo Filippi, Michael A. Foster, Antonio Gallo, Gabriel Gonzalez‐Escamilla, Cristina Granziera, Sergiu Groppa, Hanne F. Harbo, Einar A. Høgestøl, Sara Llufriu, Luigi Lorenzini, Eloy Martinez‐Heras, Silvia Messina, Marcello Moccia, Gro O. Nygaard, Jacqueline Palace, Maria Petracca, Daniela Pinter, Maria A. Rocca, Eva Strijbis, Ahmed Toosy, Paola Valsasina, Hugo Vrenken, Olga Ciccarelli, James H. Cole, Menno M. Schoonheim, Frederik Barkhof, the MAGNIMS study group, Frederik Barkhof, Nicola de Stefano, Jaume Sastre‐Garriga, Olga Ciccarelli, Christian Enzinger, Massimo Filippi, Claudio Gasperini, Ludwig Kappos, Jacqueline Palace, Menno Schoonheim, Alex Rovira, Maria Assunta Rocca, Tarek Yousry

**Affiliations:** ^1^ Queen Square Multiple Sclerosis Centre, Department of Neuroinflammation UCL Queen Square Institute of Neurology, University College London London UK; ^2^ MS Center Amsterdam, Radiology and Nuclear Medicine, Vrije Universiteit Amsterdam, Amsterdam Neuroscience, Amsterdam UMC Location VUmc Amsterdam The Netherlands; ^3^ Departments of Advanced Biomedical Sciences and Electrical Engineering and Information Technology University of Naples "Federico II" Naples Italy; ^4^ Centre for Medical Image Computing, Department of Medical Physics and Biomedical Engineering University College London London UK; ^5^ E‐Health Center, Universitat Oberta de Catalunya Barcelona Spain; ^6^ Department of Advanced Medical and Surgical Sciences University of Campania “Luigi Vanvitelli” Naples Italy; ^7^ MS Center Amsterdam, Anatomy and Neurosciences, Vrije Universiteit Amsterdam, Amsterdam Neuroscience, Amsterdam UMC Location VUmc Amsterdam The Netherlands; ^8^ Translational Imaging in Neurology (ThINK) Basel, Department of Biomedical Engineering Faculty of Medicine, University Hospital Basel and University of Basel Basel Switzerland; ^9^ Department of Neurology University Hospital Basel Switzerland; ^10^ Research Center for Clinical Neuroimmunology and Neuroscience Basel (RC2NB), university Hospital Basel and University of Basel Basel Switzerland; ^11^ Department of Neurosciences, Biomedicine and Movement Sciences University of Verona Verona Italy; ^12^ Department of Information Engineering University of Padova Padova Italy; ^13^ Department of Medicine, Surgery and Neuroscience University of Siena Siena Italy; ^14^ Department of Neurology Medical University of Graz Graz Austria; ^15^ Neuroimaging Research Unit, Division of Neuroscience, IRCCS San Raffaele Scientific Institute Milan Italy; ^16^ Neurology Unit, IRCCS San Raffaele Scientific Institute Milan Italy; ^17^ Neurorehabilitation Unit, IRCCS San Raffaele Scientific Institute Milan Italy; ^18^ Neurophysiology Service, IRCCS San Raffaele Scientific Institute Milan Italy; ^19^ Vita‐Salute San Raffaele University Milan Italy; ^20^ Movement Disorders, Neurostimulation and Neuroimaging, University Medicine Mainz Mainz Germany; ^21^ Department of Neurology Oslo University Hospital Oslo Norway; ^22^ Department of Psychology University of Oslo Oslo Norway; ^23^ Center of Neuroimmunology. Laboratory of Advanced Imaging in Neuroimmunological Diseases; Hospital Clinic Barcelona, Institut d'Investigacions Biomediques August pi i Sunyer (IDIBAPS) and Universitat de Barcelona Barcelona Spain; ^24^ Nuffield Department of Clinical Neurosciences University of Oxford Oxford UK; ^25^ Department of Neurosciences and Reproductive and Odontostomatological Sciences University of Naples “Federico II” Naples Italy; ^26^ MS Center Amsterdam, Neurology, Vrije Universiteit Amsterdam, Amsterdam Neuroscience, Amsterdam UMC Location VUmc Amsterdam The Netherlands; ^27^ Centre for Medical Image Computing, Department of Computer Science University College London London UK; ^28^ Dementia Research Centre, UCL Queen Square Institute of Neurology, University College London London UK

**Keywords:** brain connectivity, core‐periphery structure, MRI, multilayer networks, multiple sclerosis

## Abstract

Disruptions to brain networks, measured using structural (sMRI), diffusion (dMRI), or functional (fMRI) MRI, have been shown in people with multiple sclerosis (PwMS), highlighting the relevance of regions in the core of the connectome but yielding mixed results depending on the studied connectivity domain. Using a multilayer network approach, we integrated these three modalities to portray an enriched representation of the brain's core‐periphery organization and explore its alterations in PwMS. In this retrospective cross‐sectional study, we selected PwMS and healthy controls with complete multimodal brain MRI acquisitions from 13 European centers within the MAGNIMS network. Physical disability and cognition were assessed with the Expanded Disability Status Scale (EDSS) and the symbol digit modalities test (SDMT), respectively. SMRI, dMRI, and resting‐state fMRI data were parcellated into 100 cortical and 14 subcortical regions to obtain networks of morphological covariance, structural connectivity, and functional connectivity. Connectivity matrices were merged in a multiplex, from which regional coreness—the probability of a node being part of the multiplex core—and coreness disruption index (κ)—the global weakening of the core‐periphery structure—were computed. The associations of κ with disease status (PwMS vs. healthy controls), clinical phenotype, level of physical disability (EDSS ≥ 4 vs. EDSS < 4), and cognitive impairment (SDMT z‐score < −1.5) were tested within a linear model framework. Using random forest permutation feature importance, we assessed the relative contribution of κ in the multiplex and single‐layer domains, in addition to conventional MRI measures (brain and lesion volumes), in predicting disease status, physical disability, and cognitive impairment. We studied 1048 PwMS (695F, mean ± SD age: 43.3 ± 11.4 years) and 436 healthy controls (250F, mean ± SD age: 38.3 ± 11.8 years). PwMS showed significant disruption of the multiplex core‐periphery organization (κ = −0.14, Hedges' g = 0.49, *p* < 0.001), correlating with clinical phenotype (F = 3.90, *p* = 0.009), EDSS (Hedges' g = 0.18, *p* = 0.01), and SDMT (Hedges' g = 0.30, p < 0.001). Multiplex κ was the only connectomic measure adding to conventional MRI in predicting disease status and cognitive impairment, while physical disability also depended on single‐layer contributions. In conclusion, we show that multilayer networks represent a biologically and clinically meaningful framework to model multimodal MRI data, with disruption of the core‐periphery structure emerging as a potential connectomic biomarker for disease severity and cognitive impairment in PwMS.

## Graphical abstract

Using a multilayer network approach, we integrated structural, diffusion, and resting‐state functional MRI to portray an enriched representation of the connectome's core‐periphery organization in a large cohort of people with multiple sclerosis and healthy controls. We showed that the multiplex core‐periphery organization is significantly disrupted in people with multiple sclerosis, correlating with the disease phase, physical disability, and cognition.

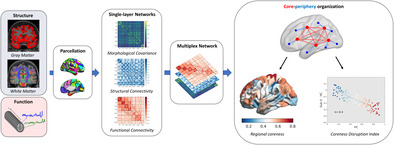

**Summary**

- Using a multilayer network approach, we integrated structural, diffusion, and resting‐state functional MRI to portray an enriched representation of the connectome's core‐periphery organization in a large cohort of people with multiple sclerosis and healthy controls
- People with multiple sclerosis show significant weakening of the multiplex core‐periphery organization compared to healthy controls, correlating with the disease phase, physical disability, and cognition
- Disruption of the multiplex core‐periphery structure is more sensitive than homologous single‐layer connectivity measures to multiple sclerosis‐related pathophysiological and cognitive changes, adding to conventional MRI measures

**Abbreviations**
BOLD: blood oxygenation level dependent
BPF: brain parenchymal fraction
CIS: clinically isolated syndrome
CSF: cerebrospinal fluid
dMRI: diffusion MRI
DMT: disease‐modifying treatment
EDSS: Expanded Disability Status Scale
FC: functional connectivity
GM: gray matter
HC: healthy controls
MC: morphological covariance
PPMS: primary‐progressive multiple sclerosis
PwMS: people with multiple sclerosis
RRMS: relapsing–remitting multiple sclerosis
rs‐fMRI: resting‐state functional MRI
SC: structural connectivity
SDMT: Symbol Digit Modalities Test
sMRI: structural MRI
SPMS: secondary‐progressive multiple sclerosis
TIV: total intracranial volume
WM: white matter

## 1 Introduction

In multiple sclerosis, there is a well‐recognized gap between clinical‐cognitive impairment and brain pathology as assessed through conventional MRI [Barkhof 2002]. The field of connectomics has now started to bridge this gap. Clinically relevant disruptions to macro‐scale brain networks, measured using structural (sMRI), diffusion (dMRI), or resting‐state functional (rs‐fMRI) MRI, have been extensively demonstrated in people with multiple sclerosis (PwMS), to the point that it has been described as a network disorder [the MAGNIMS Study Group et al. 2021].

Our current understanding points toward abnormal connectivity centred around regions such as the thalamus and the default mode network, evolving along the disease course and representing a possible mechanism through which cumulative brain damage eventually leads to long‐term disability [Pontillo et al. 2024; Schoonheim et al. 2022]. Nevertheless, MRI‐based connectivity studies often yield conflicting results, somehow failing to identify a unified connectomic hallmark of multiple sclerosis and related disability [Jandric et al. 2022]. While this is partly explained by multiple sclerosis’ intrinsic neurobiological and phenotypic heterogeneity [Pontillo et al. 2022], methodological issues may also play a role, including the disparity of image processing strategies and the small sample sizes. Moreover, one major conceptual problem lies in the focus on single‐modality networks, providing only a partial representation of the brain's complex organization.

Indeed, despite the increasing availability of multimodal neuroimaging data, most studies so far have focused on one aspect of brain connectivity using a single imaging modality (e.g., morphological covariance, MC, with sMRI; structural connectivity, SC, with dMRI; functional connectivity, FC, with rs‐fMRI) [the MAGNIMS Study Group et al. 2021]. Integrating different neuroimaging modalities into a unified brain network model holds promise to enhance our understanding of the brain and its disorders, by informing us about how structure shapes function, how they are jointly impacted by disease, and which aspects are relevant for cognitive functioning and clinical manifestations [Calhoun and Sui 2016]. The brain can be modelled as a multilayer network where different connectivity domains, each encoding a specific type of information about the system, are jointly embodied in the same topological space [De Domenico 2017]. Such topology is able to account for the simultaneous existence of different types of relationships between brain regions, potentially revealing network properties that are not evident from conventional single‐layer architectures and may be more sensitive to disease‐related changes [De Domenico 2017]. However, how to jointly model different aspects of brain connectivity is still an open challenge, with different measures that have been adopted to describe the topological properties of multilayer brain networks [Battiston et al. 2017; De Domenico, Sasai, and Arenas 2016].

In PwMS, increasing evidence suggests that the core‐periphery structure, a fundamental property of the human connectome, characterized by a subgraph of densely connected and topologically central nodes (the *core*) and a set of nodes that are strongly connected with the core but sparsely interconnected with each other (the *periphery*) [Fornito, Zalesky, and Bullmore 2016] is impacted by the disease in a clinically relevant manner [Pontillo et al. 2024; Schoonheim et al. 2022]. Indeed, connectivity alterations involving brain regions that constitute the structural and functional core of the connectome (i.e., network hubs) are a crucial event in the disease course and are most strongly associated with clinical progression [Pontillo et al. 2024; Schoonheim et al. 2022]. Nevertheless, while it has been demonstrated that the core of the human connectome can be more accurately mapped in a multilayer setting, where it encodes richer information than single‐layer aggregations [Battiston et al. 2018; De Domenico, Sasai, and Arenas 2016; Guillon et al. 2019] the impact of multiple sclerosis on the multilayer core‐periphery structure and its potential role as a biomarker of clinical severity and progression remain largely unexplored. Also, previous multimodal connectivity studies have shown that the structure–function relationship is altered in the brains of PwMS, with the integration of structural and functional information potentially enhancing our understanding of the pathophysiology and clinical correlates of the disease [Casas‐Roma et al. 2022; Kulik et al. 2022; Martí‐Juan et al. 2023; Sorrentino et al. 2022; Sorrentino et al. 2024].

Here, leveraging unique access to a large multicentric cohort of PwMS, we used a multilayer network approach to integrate information from sMRI, dMRI, and rs‐fMRI data and portray an enriched representation of the brain's core‐periphery organization. Based on previous evidence that network hubs are prominently affected in PwMS, we hypothesised that joint brain network changes across structural and functional levels would manifest in a disrupted multilayer core‐periphery structure compared to healthy individuals. A multilayer analysis is expected to be more sensitive to multiple sclerosis‐related pathophysiological alterations and enable more accurate predictions of physical and cognitive disability compared to unimodal approaches.

## 2 Materials and Methods

### 2.1 Participants

In this retrospective, cross‐sectional study, we collected MRI and clinical data of people diagnosed with multiple sclerosis according to 2010 McDonald criteria [Polman et al. 2011] or clinically isolated syndrome (CIS) [Lublin et al. 2014] from 13 European centers (MAGNIMS: www.magnims.eu). Healthy controls (HC) without a history of neurologic or psychiatric disorders were also included. At the time of MRI, PwMS were clinically evaluated using the Expanded Disability Status Scale (EDSS) [Kurtzke 1983] and the Symbol Digit Modalities Test (SDMT) [Benedict et al. 2017], measuring physical disability and cognition, respectively. Raw SDMT scores were transformed to age‐, sex‐, and education‐adjusted z‐scores according to population‐specific normative data [Amato et al. 2006; Eijlers et al. 2019; Scherer et al. 2004; Sepulcre et al. 2006; Strober et al. 2020].

Written informed consent had been obtained from each participant independently at each center. The final protocol for this study was reviewed and approved by the local Ethics Committee and the European MAGNIMS collaboration for the analysis of pseudonymized data.

### 2.2 MRI Data Acquisition and Processing

**FIGURE 1:**
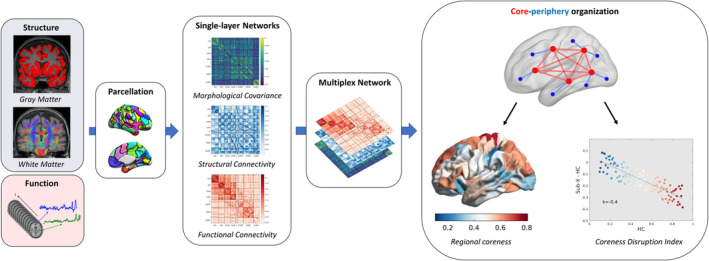
Schematic illustration of the analysis pipeline. SMRI, dMRI, and rs‐fMRI are processed using the same brain parcellation scheme to obtain networks of morphological covariance, structural connectivity, and functional connectivity, respectively. Connectivity matrices are then merged in a multiplex network, a particular case of multilayer network where there is a one‐to‐one correspondence between nodes at different layers. The multiplex core‐periphery organization is characterized in terms of regional coreness, defined as the probability of a node being part of the multiplex core and coreness disruption index (κ), quantifying the global weakening of the core‐periphery structure.

All participants were imaged on 3 T scanners with a brain MRI protocol including isotropic T1‐weighted (T1w), T2‐weighted fluid‐attenuated inversion recovery (FLAIR), dMRI, and RS‐fMRI sequences. Details of the different acquisition protocols are provided in Supplementary Table 1, while a schematic illustration of the analysis pipelines discussed below is shown in Figure 1.

#### 2.2.1 Structural MRI and Morphological Covariance Networks

For PwMS, T2‐hyperintense lesions were automatically segmented on FLAIR images using the Lesion Segmentation Tool (LST) 3.0.0 (www.statistical‐modelling.de/lst.html). Corresponding masks were used to fill lesions in T1w images with estimated white matter (WM) tissue for subsequent analyses [Chard et al. 2010] and to compute total lesion volume (TLV). We used the Computational Anatomy Toolbox (CAT12.7, http://www.neuro.uni‐jena.de/cat) to segment T1w volumes into grey matter (GM), WM, and cerebrospinal fluid (CSF), and to parcellate the brain into 100 cortical regions from the Schaefer atlas (https://github.com/ThomasYeoLab/CBIG/tree/master/stable_projects/brain_parcellation/Schaefer2018_LocalGlobal) [Schaefer et al. 2018]. This functional parcellation is designed to optimize both local gradient and global similarity measures of the fMRI signal [Schaefer et al. 2018]. The nodes are also associated with 7 canonical functional system labels including visual, somatomotor, dorsal attention, ventral attention, limbic, control, and default mode networks [Thomas Yeo et al. 2011]. We chose the 100‐parcel version to best fit the spatial resolution of the available data (Supplementary Table 1). In addition, we used FSL‐FIRST to segment 14 subcortical GM regions [Patenaude et al. 2011]. Throughout the diffusion and functional workflows, T1w images were used as reference and underwent additional processing steps, including cortical surface reconstruction with recon‐all (FreeSurfer v6.0.1) [Dale, Fischl, and Sereno 1999].

Single‐subject GM networks were obtained by adapting a previously described pipeline [Jy et al. 2020]. Briefly, the volumes of the 114 atlas‐defined GM regions were transformed into z‐scores while adjusting for the physiological (i.e., estimated in the HC group) effects of age, sex and total intracranial volume (TIV), and a 114 x 114 MC matrix was obtained where the following measure of shared deviation from the reference norm represented the edge weights (distributed between 0 and 1).

Joint variation between the *i‐th* (for *i* = 1 to 114) and *j‐th* (for *j* = 1 to 114) GM regions = 1/exp.{[(z‐transformed volume of *i‐th* region) – (z‐transformed value of *j‐th* region)]^2^}.

#### 2.2.2 Diffusion MRI and Structural Connectivity Networks

Preprocessing of diffusion MRI data was performed using QSIPrep 0.14.3 [Cieslak et al. 2021], which is based on Nipype 1.6.1 [Gorgolewski et al. 2011]. MP‐PCA denoizing as implemented in MRtrix3's dwidenoise was applied with a 5‐voxel window, followed by B1 field inhomogeneity correction using dwibiascorrect from MRtrix3 with the N4 algorithm [Tustison et al. 2010] FSL (version 6.0.3) eddy was used to correct for head motion and eddy currents [Andersson and Sotiropoulos 2016]. A deformation field to correct for susceptibility distortions was estimated using available sequences (phase‐encoding polarity method [Jezzard and Balaban 1995], phase‐difference B0 estimation [Hutton et al. 2002], or registration‐based fieldmap‐less estimation [Wang et al. 2017]) and used to calculate an unwarped b = 0 reference for a more accurate co‐registration with the anatomical reference. The diffusion‐weighted time‐series was then resampled to the T1w volume, producing a preprocessed diffusion‐weighted series with 2 mm isotropic voxels. Then, multi‐tissue fiber response functions were generated using the Dhollander algorithm [Dhollander et al. 2019], and fiber orientation distributions (FODs) were estimated via constrained spherical deconvolution and intensity‐normalized using mtnormalize [Raffelt et al. 2017; Tournier, Calamante, and Connelly 2007]. Tractography was performed based on WM FODs with MRtrix3's tckgen, using the iFOD2 probabilistic tracking method to generate 10 million streamlines, with anatomical constraints provided by a hybrid surface/volume segmentation created ad hoc [Smith et al. 2012; Smith et al. 2020].

Finally, weights for each streamline were calculated using SIFT2 [Smith et al. 2015] and a 114 x 114 SC matrix was filled with the sums of weights of streamlines connecting each node's pair. In addition, structural connectivity matrices were log_10_‐transformed to better account for differences at different magnitudes and to make the distribution of edges' weight more comparable to other layers [Buchanan et al. 2020].

#### 2.2.3 Resting‐State Functional MRI and Functional Connectivity Networks

Preprocessing of rs‐fMRI data was performed using fMRIPrep 20.2.6 [Esteban et al. 2019] which is based on Nipype 1.7.0 [Gorgolewski et al. 2011]. From blood oxygenation level dependent (BOLD) data, a reference volume and its skull‐stripped version were generated using a custom methodology of fMRIPrep. Similar to dMRI processing, a deformation field to correct for susceptibility distortions was estimated based on available sequences and used to calculate a corrected EPI reference for a more accurate co‐registration with the anatomical reference. The BOLD reference was then co‐registered to the T1w reference using bbregister (FreeSurfer) which implements boundary‐based registration [Greve and Fischl 2009]. Head‐motion parameters for the BOLD reference were estimated before any spatiotemporal filtering using mcflirt (FSL 5.0.9) [Jenkinson et al. 2002], After slice‐timing correction using 3dTshift from AFNI 20160207 [Cox 1996], the BOLD time‐series were resampled onto their original, native space by applying a single, composite transform to correct for head‐motion and susceptibility distortions. Several confounding time‐series were calculated based on the preprocessed BOLD, including the identification of noise components using ICA‐AROMA [Pruim et al. 2015]. Nuisance variables were removed from preprocessed BOLD using Nilearn 0.8.1 [Abraham et al. 2014], following a previously described strategy [Chai et al. 2012] including removal of the first 4 timepoints, band‐pass filter (0.008–0.08 Hz), detrending, standardization and confound regression (nonaggressive ICA‐AROMA denoising plus removal of mean WM and CSF signal) [Pruim et al. 2015].

Finally, residual mean BOLD time series were obtained from the atlas‐defined parcels, and, for each node's pair, the Pearson correlation coefficient was computed and Fisher z‐transformed to fill a 114 x 114 FC matrix. In addition, matrices were absolutized as inverse correlations may encode relevant information and most analysis strategies tend to neglect negative values [Chai et al. 2012].

#### 2.2.4 Quality Control and Cross‐Site Harmonization

MRI quality was assessed through metric‐guided visual inspection. Scans that were marked as outliers (i.e., outside 1.5 times the interquartile range in the adverse direction of the measurement distribution) according to one or more image quality metrics obtained via CAT12 (for sMRI), qsiprep (for dMRI), and mriqc 0.16.1 (for sMRI and rs‐fMRI) [Esteban et al. 2017] were reviewed by a single investigator, a neuroradiologist with ten‐year experience in advanced neuroimaging (G.P.), and discarded based on visual evaluation where appropriate. The number of excluded scans per site is provided in Supplementary Table 1.

To eliminate nonbiological site‐related variability, we used ComBat harmonization to model and remove site effects from brain volumes and structural and functional connectivity matrices, while preserving the biological associations with age, sex, and disease status (PwMS vs. HC) [Johnson, Li, and Rabinovic 2007].

### 2.3 Multiplex Networks and Core‐Periphery Organization

To correct for differences in average link weight across layers, MC, SC, and FC connectivity matrices underwent singular‐value decomposition normalization before the construction of a multimodal multiplex, a particular case of multilayer network where there is a one‐to‐one correspondence between nodes at different layers [Mandke et al. 2018]. For each brain region, we extracted coreness using the brain network toolbox (https://github.com/brain‐network/bnt), following a previously described procedure [Battiston et al. 2018]. Briefly, each layer is filtered by preserving the strongest weights for the full range of density‐based thresholds. At each threshold, a measure of node richness in the multiplex setting is computed by linearly combining node strengths in all layers through a vector of coefficients modulating the relative importance of each layer. As we did not know a priori which connectivity domain would be more relevant for the explored experimental settings, we imposed equal coefficients (0.5) for the MC, SC, and FC layers, as in *Battiston et al* [Battiston et al. 2018].

Multiplex richness is then fed into a core‐periphery decomposition procedure, and coreness is calculated as the number of times that each node is present in the network core across all explored thresholds, normalized by the maximum theoretical value (i.e., the total number of explored thresholds) [Battiston et al. 2018]. Coreness disruption index (κ) was computed as the slope of the linear regression model between the mean local coreness of the HC group at each node, taken as a reference, and the differential nodal coreness between that reference and the subject(s) under study [Termenon et al. 2016].

### 2.4 Statistical Analysis

Second‐level analyses were carried out using MATLAB (R2020a). Significance level was set at α = 0.05 for all tests, adjusting for multiple comparisons when appropriate. The effects of biological confounders (i.e., age and sex) on variables of interest (i.e., regional coreness and coreness disruption index) were removed using nuisance regression, with weights estimated in the HC group to avoid removing disease‐related variance. Specifically, for each measure, a linear model with the confounders as independent variables was fit in the HC group and used to generate predictions on the whole population. Confounder‐adjusted measures were obtained as the difference between raw and predicted values, and standardized as follows: individual z‐score = (individual corrected value—mean of corrected values in the HC group) / standard deviation of corrected values in the HC group.

Differences between patients and HC in terms of κ and nodal coreness (over the full set of brain parcels) were assessed using two‐sided permutation t tests, controlling for the false discovery rate with the Benjamini–Hochberg procedure [Benjamini and Hochberg 1995]. Additionally, to assess possible κ differences across clinical phenotypes (CIS vs. relapsing–remitting, RRMS vs. secondary‐progressive, SPMS vs. primary‐progressive, PPMS), we performed a one‐way ANOVA analysis, with post hoc tests using Tukey's method [Field et al. 2012]. The associations between κ and levels of physical (EDSS ≥ 4 vs. EDSS < 4) [Confavreux and Vukusic 2006] and cognitive (impaired, SDMT z‐score < −1.5, vs. preserved information processing speed, IPS) [Eijlers et al. 2019] disability were assessed using two‐sided permutation t tests.

To demonstrate that the findings were not driven by contingent factors including parcellation scheme/inaccuracy and site effects/harmonization, we conducted a set of sensitivity analyses. First, we replicated the analyses using an alternative cortical parcellation based on the Brainnetome atlas, providing a more fine‐grained definition of functional brain subregions (210 cortical and 14 subcortical parcels) [Fan et al. 2016]. Also, as brain atrophy is known to potentially affect the accuracy of atlas‐based parcellations, we conducted a subset analysis on participants with relatively preserved global brain volume (age‐ and sex‐adjusted brain parenchymal fraction, BPF, z‐score based on the distribution in HC > −1.5). Finally, we used the largest cohort for a single‐site analysis on nonharmonized matrices.

To evaluate the added value of our multiplex approach over single‐layer connectomic metrics, we computed κ within the MC, SC, and FC layers separately and looked at the ability of measures in different domains to discriminate between PwMS and HC, as well as between different levels of physical and cognitive disability. Comparison was made also with other established conventional MRI measures of multiple sclerosis‐related brain damage (i.e., age‐ and sex‐adjusted brain parenchymal fraction, BPF, and total lesion volume, TLV), used as a reference. First, we compared effect sizes (Hedges' g) of the between‐groups differences for the different MRI‐derived variables, by computing 95% bootstrap confidence intervals with 5000 resamples [Durlak 2009] Also, to assess whether connectome‐based metrics added to BPF and TLV, we used κ in the different domains and conventional MRI measures to train and validate random forest models for the prediction of disease status (PwMS vs. HC), level of physical disability, and IPS impairment. Specifically, decision tree learners were combined with bootstrap aggregation, and relevant hyperparameters were tuned using Bayesian optimization in order to minimize the 10‐fold CV classification error (1—accuracy) [Breiman 2001]. Model performance was expressed with out‐of‐bag (OOB) accuracy, while the relative weight of different predictors was estimated using OOB permutation feature importance [Breiman 2017].

## 3 Results

### 3.1 Participants

A total of 1517 participants were considered for this study. Of these, 33 were excluded due to poor MRI quality or image processing failure, leading to a final population including 1048 PwMS and 436 HC. Demographic, clinical, and conventional MRI characteristics of the studied population are reported in Table 1.

**TABLE 1:** Demographic, clinical, and MRI characteristics of the studied population.

	PwMS	HC
N	1048	436
Age [yr], mean ± SD	43.3 ± 11.4	38.3 ± 11.8^a^
Female, n (%)	695 (66.3)	250 (57.3)^b^
Disease duration [yr], mean ± SD	11.6 ± 8.9	—
Phenotype, n (%):		—
CIS	41 (3.9)	
RRMS	817 (78.0)	
SPMS	121 (11.5)	
PPMS	69 (6.6)	
EDSS, median (range)	2.5 (0.0–8.0)	—
SDMT [z‐score], mean ± SD	−0.77 ± 1.36	—
DMT, n (%): yes/no	926 (88.4) / 122 (11.6)	—
TLV [ml], median (interquartile range)	3.14 (1.06–8.00)	—
BPF, mean ± SD	0.77 ± 0.05	0.80 ± 0.03^c^

Abbreviations: BPF = brain parenchymal fraction; CIS = clinically isolated syndrome; DMT = disease‐modifying treatment; EDSS = Expanded Disability Status Scale; HC = healthy controls; PPMS = primary‐progressive multiple sclerosis; PwMS = people with multiple sclerosis; RRMS = relapsing–remitting multiple sclerosis; SD = standard deviation; SPMS = secondary‐progressive multiple sclerosis; SDMT = Symbol Digit Modalities Test; TLV = total lesion volume.

^a^ *p* < 0.001 (permutation t test).

^b^ *p* = 0.001 (Chi‐square test).

^c^ *p* < 0.001 (permutation t test).

### 3.2 Multiplex Networks and Core‐Periphery Organization

SMRI, dMRI, and rs‐fMRI data were processed using the same brain parcellation scheme (including 100 cortical and 14 subcortical regions) to obtain networks of MC, SC, and FC, respectively. Average connectivity matrices in the HC group are shown in Supplementary Figure 1.

**FIGURE 2:**
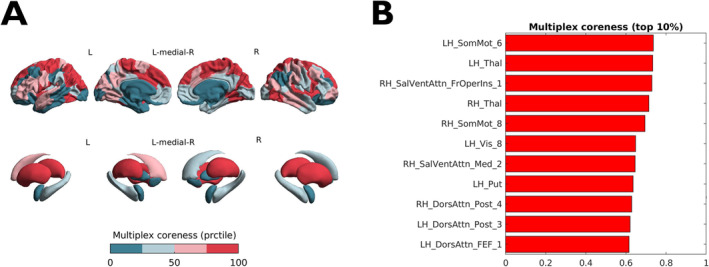
Average multiplex coreness in the healthy controls group. (A) Color‐coded (*teal* to *red*) map of multiplex coreness percentile ranks superimposed on surface renderings of the cortex and subcortical structures. Image was obtained with the ENIGMA toolbox [Larivière et al. 2021]. (B) Highest 10% multiplex coreness nodes and corresponding absolute values are shown. Nomenclature of cortical areas follows the 7‐network Schaefer‐100 parcellation [Schaefer et al. 2018].

In the HC group, the multiplex core included on average subcortical GM structures, especially the thalami and putamina, as well as cortical areas participating in both sensorimotor (somatomotor and visual) and associative (default mode, control, and attention) networks (Figure 2). Maps of average HC regional coreness in the single‐layer domains are shown in Supplementary Figure 2.

### 3.3 Disrupted Multiplex Core‐Periphery Structure in Multiple Sclerosis

**FIGURE 3:**
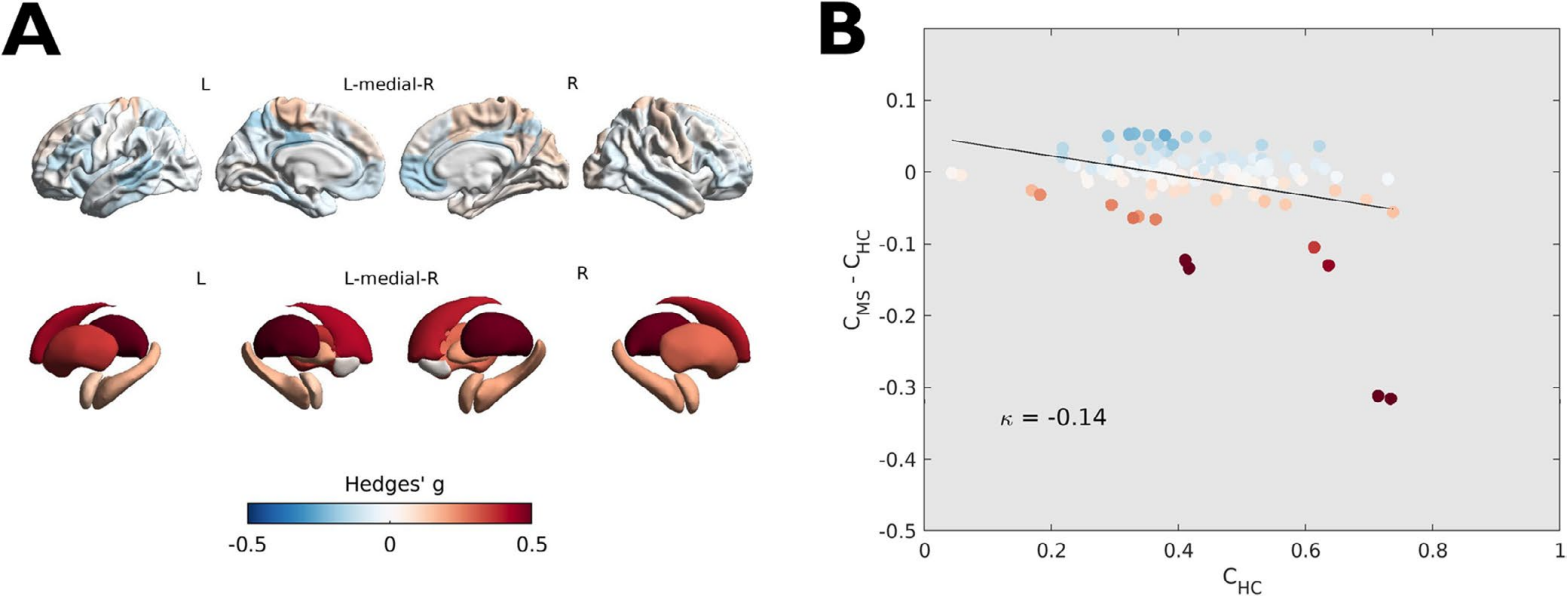
Differences in regional coreness between PwMS and HC and coreness disruption index. (A) Color‐coded (*blue* to *red*) map of effect sizes (Hedges' g) of the between‐group difference superimposed on surface renderings of the cortex and subcortical structures. Image was obtained with the ENIGMA toolbox [Larivière et al. 2021]. (B) Scatterplot showing, region‐wise, the between‐group difference in average regional coreness as a function of the average coreness in the HC group. The slope of the linear regression line corresponds to the coreness disruption index κ = −0.14. Each circle represents a brain region, color‐coded as in panel A.

PwMS showed significant deviations in regional coreness compared to the HC group (Figure 3A), with the greatest effect sizes observed at the level of deep GM structures (reduced coreness) and associative areas in the medial prefrontal, cingulate, and lateral temporal cortices (increased coreness) (Supplementary Table 2).

Based on the profile of regional coreness' deviations from the healthy norm, coreness disruption index (κ) was computed as a measure of global weakening of the brain's core‐periphery structure. In PwMS, the anatomical distribution of the observed changes was such that topologically central nodes were generally more impacted than peripheral ones (which tended to have preserved or even increased coreness values), as expressed by the average κ = −0.14 (Hedges' g = 0.49, *p* < 0.001) (Figure 3B).

**FIGURE 4:**
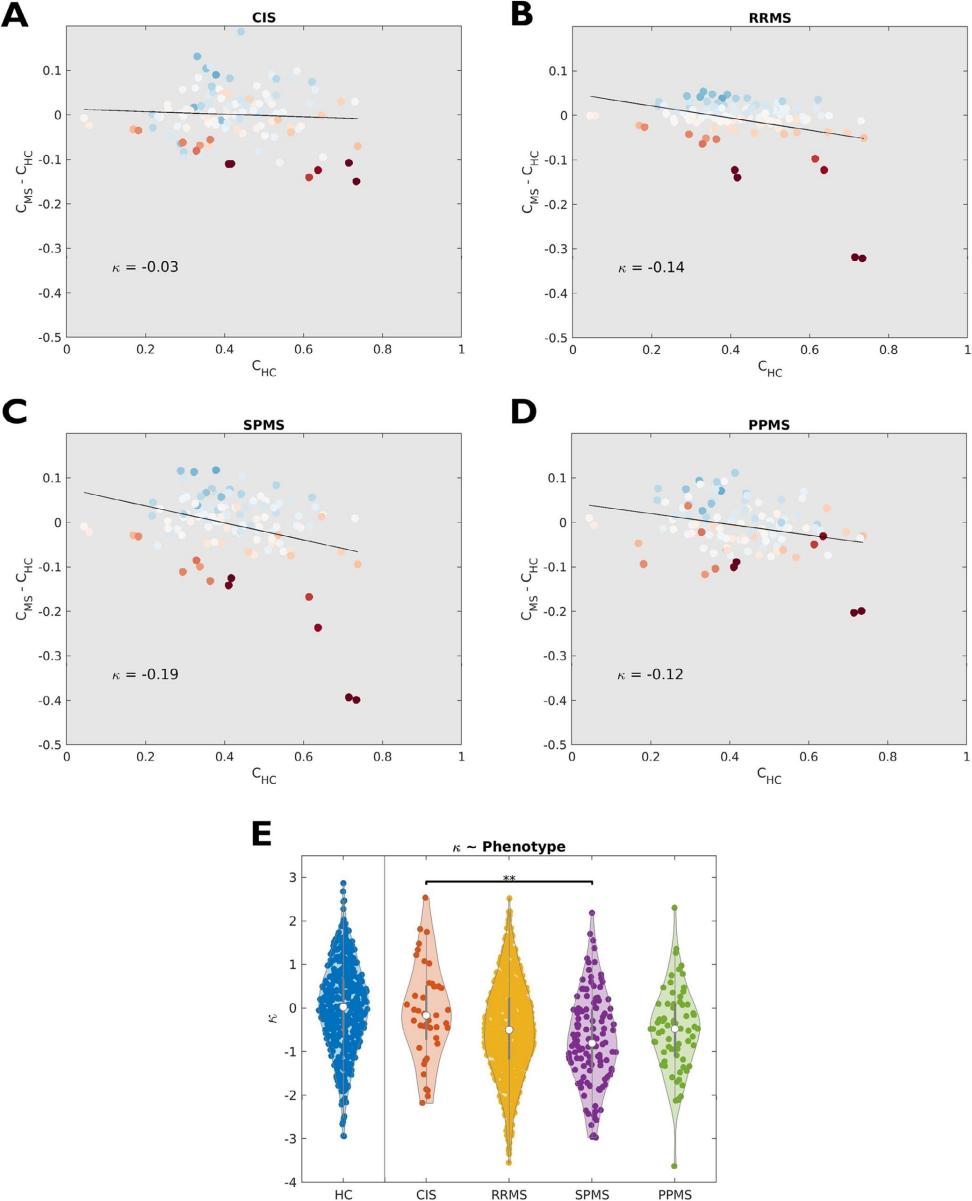
Coreness disruption index and clinical phenotypes. Coreness disruption index (κ) plots are shown for (A) clinically isolated syndrome (CIS), (B) relapsing–remitting (RRMS), (C) secondary‐progressive (SPMS), and (D) primary‐progressive (PPMS) patients. (E) Violin plots showing the distributions of κ values, expressed as confounder‐adjusted z‐scores, across different phenotypes. The distribution in healthy controls is also shown for comparison. (**) Adjusted *p* < 0.01. In (A‐D), for each subgroup, the difference in average regional coreness compared to HC is plotted as a function of the average coreness in the HC group. Each circle represents a brain region, with color encoding the magnitude of the between‐group (MS/CIS versus HC) difference in terms of regional coreness.

**FIGURE 5:**
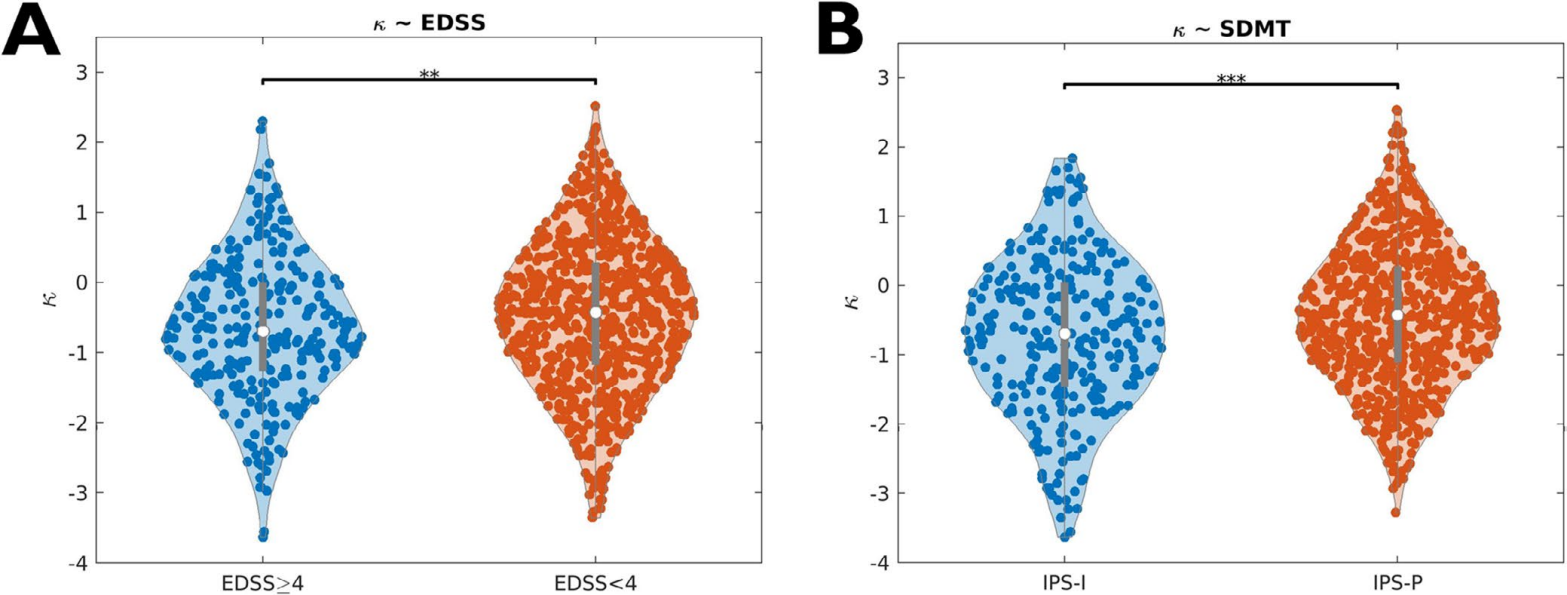
Coreness disruption index and clinical variables. Violin plots showing the distribution of confounder‐adjusted coreness disruption index (κ) z‐scores in patients with (A) high (EDSS ≥ 4) and low (EDSS < 4) levels of physical disability, and (B) impaired (IPS‐I) and preserved (IPS‐P) information processing speed at the SDMT. (**) *p* < 0.01; (***) *p* < 0.001. EDSS = Expanded Disability Status Scale; SDMT = Symbol Digit Modalities Test.

There was a significant effect of clinical phenotype on the weakening of the core‐periphery structure of multimodal brain networks (F[3, 1044] = 3.90, *p* = 0.009). We observed, on average, progressively greater disruption in relapse‐onset forms going from CIS to SPMS patients and intermediate κ values in PPMS (Figure 4). Moreover, when classifying PwMS according to levels of physical disability (EDSS ≥ 4, 27% vs. EDSS < 4, 73%) [Confavreux and Vukusic 2006] and IPS (impaired, IPS‐I, 29% vs. preserved, IPS‐P, 71%) [Eijlers et al. 2019], a stronger/less disrupted core‐periphery organization was associated with both lower physical disability (Hedges' g = 0.18, *p* = 0.01) and preserved cognition (Hedges' g = 0.30, *p* < 0.001) (Figure 5).

A set of sensitivity analyses substantially replicated these findings (Supplementary Results), demonstrating their relative robustness to the choice of the parcellation scheme (Supplementary Figures 3–4), brain atrophy (Supplementary Figure 5), and site effects/harmonization (Supplementary Figure 6). Indeed, the effect sizes for the coreness disruption index at the PwMS vs HC comparison (Hedges' g ranging from 0.40 to 0.63 vs 0.49 in the main analysis) and the associations with physical disability (Hedges' g ranging from 0.16 to 0.32, vs 0.18 in the main analysis) and IPS (Hedges' g ranging from 0.27 to 0.38 vs 0.30 in the main analysis) were comparable to those observed in the main analysis.

### 3.4 Added Value of Multiplex Over Single‐Layer Network Measures

Correlations between κ values of PwMS in the multiplex and single‐layer domains, along with their distributions, are depicted in Supplementary Figure 7.

**FIGURE 6:**
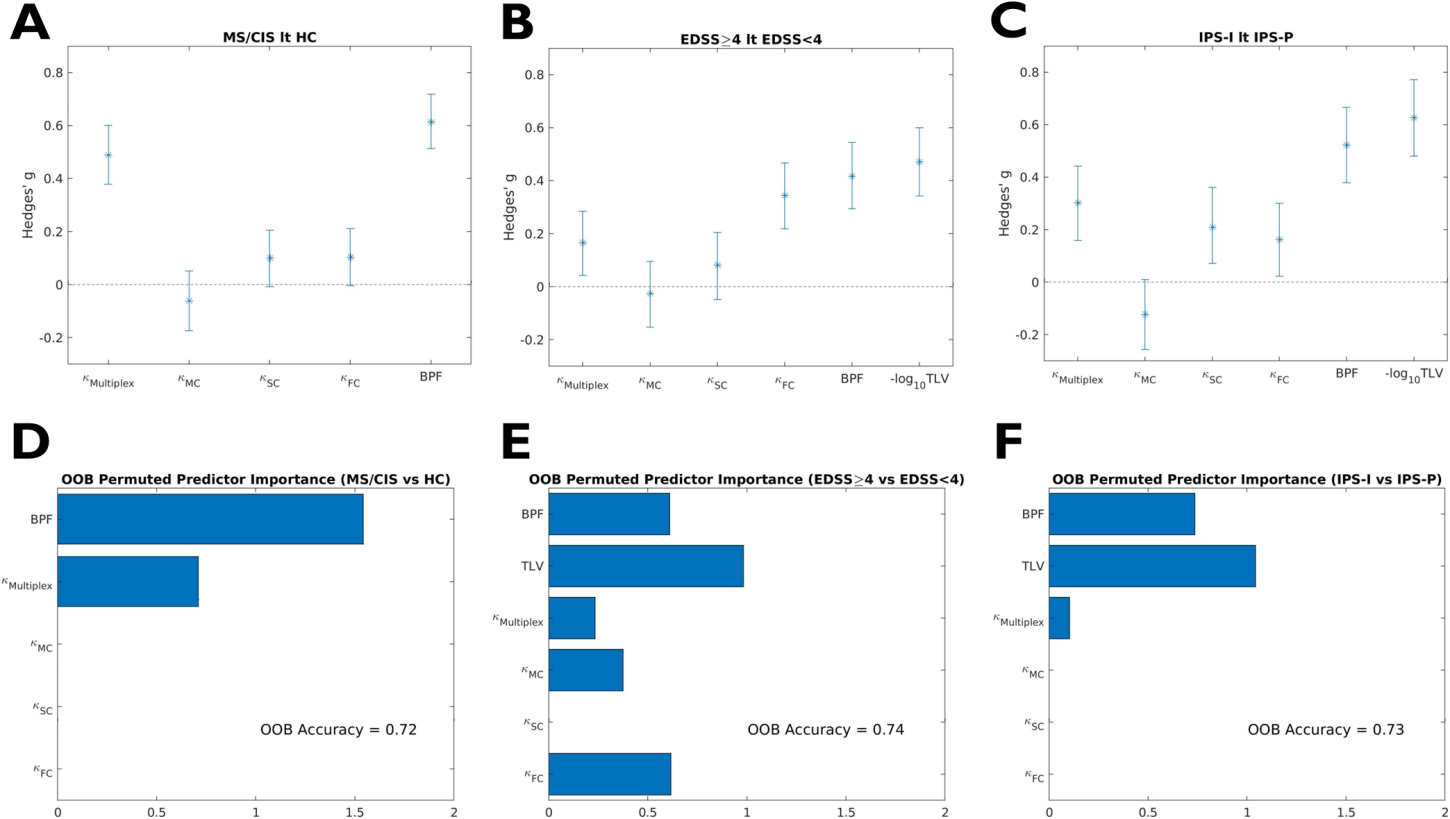
Added value of multiplex over single‐layer measures. For indices of coreness disruption in the multiplex and single‐layer domains, as well as for brain parenchymal fraction (BPF) and total lesion volume (TLV), shown are (*top row*) the effect sizes (Hedges' g) and corresponding 95% confidence intervals associated with differences between (A) PwMS and healthy controls (MS/CIS vs. HC), (B) patients with high and low levels of physical disability (EDSS ≥ 4 vs. EDSS < 4), and (C) patients with impaired and preserved information processing speed (IPS‐I vs. IPS‐P); (*bottom row*) results of the random forest classifiers and corresponding predictor importance analyses for the prediction of (D) disease status (MS/CIS vs. HC), (E) level of physical disability (EDSS ≥ 4 vs. EDSS < 4), and (F) impaired information processing speed (IPS‐I vs. IPS‐P).

Disease status (CIS/MS vs. HC) and IPS performance (IPS‐I vs. IPS‐P) were more strongly associated with disruption of the core‐periphery structure in the multiplex setting (Hedges' g = 0.49 [95% CI = 0.38–0.60] and 0.30 [95% CI = 0.16–0.44], respectively) than with any of the single‐layer measures. As for the level of physical disability (EDSS ≥ 4 vs. EDSS < 4), the effect size associated with the disruption of the multiplex core‐periphery organization (Hedges' g = 0.17 [95% CI = 0.04–0.29]) was not significantly higher than for homologous single‐layer measures (Figure 6A–C).

Random forest models leveraging both conventional volumetric and global core‐periphery organization‐related MRI measures reached out‐of‐sample accuracies of 0.72, 0.74 and 0.73 for the CIS/MS vs. HC, EDSS ≥ 4 vs. EDSS < 4 and IPS‐I vs. IPS‐P classifications, respectively. For both disease and cognitive status predictive models, disruption of the core‐periphery structure in the multiplex domain was the only connectomic metric independently contributing to the classification along with MRI‐derived volumes. On the other hand, core‐periphery disruption in single‐layer domains (i.e., MC and FC) were at least as important as the homologous multiplex measure in predicting the level of physical disability (Figure 6D–F).

## 4 Discussion

By jointly modelling three different MRI modalities in a multilayer framework, we revealed clinically relevant disruption of the core‐periphery organization of multimodal (structural‐functional) brain networks in a large multicentric sample of PwMS. We showed that the degree of weakening of the multiplex core‐periphery depends on the disease phase and is associated with physical disability and cognition, being more sensitive than homologous single‐layer connectivity measures to multiple sclerosis‐related pathophysiological and cognitive changes and adding to conventional MRI measures.

As the information conveyed by connectivity data is multivariate in nature and multimodal datasets become increasingly available, it has been advocated that multilayer networks, rather than single‐layer architectures, may represent the ideal mathematical framework to study the brain as a complex system [Battiston et al. 2018; Casas‐Roma et al. 2022; De Domenico 2017]. However, how to meaningfully model together structural and functional aspects of brain connectivity is still debated, with novel possible methodological solutions continuing to emerge [Battiston et al. 2018; Casas‐Roma et al. 2022; De Domenico 2017]. The method adopted here to detect the core‐periphery of multiplex networks has the advantage of minimizing the need for a priori assumptions, reducing the variable degree of arbitrariness and information loss that are inevitably associated with the processes of, e.g., thresholding/binarizing connectivity matrices, or explicitly modelling interlayer links [Battiston et al. 2018].

In keeping with previous knowledge on the structural and functional cores of the human connectome [Bassett et al. 2013; Hagmann et al. 2008] average coreness maps in the HC group revealed that the SC core included the superior frontal and superior parietal cortex, as well as subcortical GM structures, while rolandic and occipital cortical regions participating in the somatomotor and visual networks constituted the functional core. On the other hand, a less pronounced core‐periphery organization was observed in the MC layer, with lower absolute values of core nodes resulting from a more distributed coreness pattern. Notably, while the coreness of the multiplex network was strongly influenced by the SC layer, it also captured the role of functional hubs (e.g., in the occipital cortices) whose importance was neglected by diffusion‐based networks. This confirms that, while being sensitive to single‐layer contributions, the multiplex setting provides a unique, and potentially more accurate, representation of the brain core‐periphery structure [Battiston et al. 2018].

In PwMS, the regional coreness profile deviated significantly from the control group, with some increases in the associative cortex, a prominent decrease in subcortical GM structures, and the greatest effect sizes observed at the level of the thalami. The thalamus is widely recognized as a vulnerable site for multiple sclerosis‐related damage, with atrophy, structural disconnection and functional reorganization occurring from the early stages, evolving with the disease course and driving disability progression and cognitive impairment [Schoonheim et al. 2015]. Hence, it is not surprising to observe how thalamic structural and functional modifications result in a reduced topological centrality in the multiplex setting. Also, the increased centrality of associative areas implicated in the default mode, control, and dorsal attention networks can be interpreted as a manifestation of network reorganization and (functional) rerouting phenomena [Eijlers et al. 2017; Schoonheim et al. 2022].

At the global level, multiple sclerosis was associated with the weakening of the multiplex core‐periphery structure, with hub regions found to be more impacted than would be expected based solely on their reference coreness in non‐diseased subjects. From a network science perspective, this conceptually equates multiple sclerosis with a targeted (i.e., network elements are impacted according to some index of topological centrality), rather than a random (i.e., network elements are impacted with uniform probability), attack [Fornito, Zalesky, and Breakspear 2015]. Previous evidence from sMRI [Steenwijk et al. 2016], dMRI [Shu et al. 2018], and fMRI [the MAGNIMS Study Group et al. 2021] studies suggested that multiple sclerosis‐related brain damage occurs in a nonrandom, network‐mediated fashion, a hypothesis that bears great transdiagnostic relevance as it seems to apply to many different neurological and psychiatric disorders [Cauda et al. 2018]. Several mechanisms (not necessarily mutually exclusive) have been proposed to explain this phenomenon, including diaschisis/transneuronal degeneration, nodal stress, shared vulnerability, and propagation of toxic agents/neuroinflammatory response along neuronal connections [Chard and Miller 2016; Jandric et al. 2021]. Our multimodal analysis suggests that these processes are likely to impact the different layers of brain connectivity in a synergistic manner, as witnessed by the greater sensitivity of the multimodal network model toward multiple sclerosis‐related changes compared to single‐layer architectures. Indeed, a disruption of the core‐periphery structure in PwMS was also evident in the SC and FC layers alone, correlating with multiplex κ values. These are also the connectivity domains with a more pronounced core‐periphery organization in the reference HC group, whose disruption falls in line with the known predilection of multiple sclerosis‐related brain damage for structural and functional network hubs [Pontillo et al. 2024; Schoonheim et al. 2022]. On the other hand, a more preserved/stronger core‐periphery organization in PwMS was observable in the MC domain, which might be the expression of a more distributed GM damage [Collorone et al. 2020]. Interestingly, while correlating with alterations in the SC and FC layers, disruption of the core‐periphery organization appeared most prominently in the multiplex setting, despite the slight opposite contribution of the MC layer. This finding confirms that a multilayer framework can encode richer information on disease‐related changes to the brain's core‐periphery organization than single‐layer approaches.

While our sample was largely composed of patients with relapsing–remitting multiple sclerosis, an association between clinical phenotype and weakening of the core‐periphery structure was still observable. A linear trend of progressive disruption in relapse‐onset forms suggesting that it may parallel the progression of brain damage along the disease course. Also, disruption of the multiplex core‐periphery organization was significantly associated with levels of physical and cognitive disability, supporting the clinical relevance of the observed connectomic changes.

Core‐periphery disruption in multimodal, rather than single‐layer, networks contributed to the prediction of cognitive impairment, adding to conventional MRI measures. This confirms that the information conveyed by multilayer networks is more than just the sum of its parts, capturing network properties that are relevant for cognition but not evident from single layers (alone or in combination). On the other hand, a noisier picture emerged for the prediction of physical disability, although the relatively limited reliability of EDSS as a method to quantify disability and the contribution of spinal cord damage (unexplored here) are likely to play an important role [Meyer‐Moock et al. 2014]. While simpler volumetric measures were still determinant for clinical predictions, we observed that a single comprehensive connectivity‐based measure (multiplex κ) was associated with multiple sclerosis almost as strongly as whole brain volume, and consistently added to conventional MRI metrics for the prediction of disease status and cognition, supporting its potential as a clinically relevant biomarker of connectome disruption.

The present study is not without limitations. First, the proposed approach is only one of the many possible solutions to model multivariate brain connectivity data, with alternative methods that may be more appropriate according to the research question and the available data or resources. Also, the purely cross‐sectional nature of our dataset limits the potential for investigating causal relationships and exploring the prognostic value of the observed connectomic changes. In addition, clinical evaluations were limited to only EDSS and SDMT, whose weaknesses from a clinimetric point of view were exacerbated by the heterogeneity of the studied population and the retrospective, multisite, multi‐country nature of our study [Goldman et al. 2019]. To reliably estimate brain–behaviour associations in this setting, along with the efforts to improve neuroimaging data acquisition and processing, attention should be drawn on the clinical side of the equation to quantify disability in a more refined manner by, e.g., assessing additional cognitive domains and specific motor functions, or adopting specific denoising/harmonization strategies [Tiego and Fornito 2022]. Finally, we used multilayer networks with the mainly descriptive purpose of characterizing brain connectivity modifications and their clinical correlates in multiple sclerosis. Future studies following a predictive approach will be needed to validate the proposed neuroimaging‐based biomarker as clinically useful beyond conventional measures and drive the analysis of multimodal connectivity data towards translational clinical impact [Woo et al., 2017].

## 5 Conclusion

In conclusion, we show that multilayer networks represent a biologically and clinically meaningful framework to jointly model multimodal MRI data, with disruption of the core‐periphery structure emerging as a potential biomarker for disease severity and cognitive impairment in multiple sclerosis.

## Author Contributions

G.P., F.P., A.M.W., J.H.C., M.M.S., and F.B. conceived and planned the experiments and contributed to the interpretation of the results. G.P. carried out the experiments with support from F.P., A.M.W., and B.K. G.P. took the lead in writing the manuscript. F.B. supervised the project. All authors contributed to data collection, provided critical feedback and helped shape the manuscript.

## Conflicts of Interest

Linda Douw and Massimo Filippi are handling editors of human brain mapping and co‐authors of this article. To minimize bias, they were excluded from all editorial decision‐making related to the acceptance of this article for publication. G.P. was supported by the ECTRIMS‐MAGNIMS Research Fellowship Programme (2020). F.P. and B.K. are supported by the UK National Institute for Health Research (NIHR) Biomedical Research Centre (BRC) at UCLH and UCL. A.C. is supported by EUROSTAR E!113,682 HORIZON2020. M.C. received speaker honoraria from Biogen, Bristol Myers Squibb, Celgene, Genzyme, Merck Serono, Novartis, and Roche and receives research support from the Progressive MS Alliance and Italian Minister of Health. Sa.Co. is supported by a Rosetrees Trust Grant (PGL21/10079). M.A.F. is supported by a grant from the MRC (MR/S026088/1). Si.Co. serves on scientific advisory board for Amicus Therapeurics has received speaker honoraria from Sanofi and research grants from Fondazione Italiana Sclerosi Multipla and Telethon. R.S. was awarded a MAGNIMS‐ECTRIMS fellowship in 2019. L.D. Is supported by the Dutch Research Council (NWO, Vidi 198.015). M.F. is Editor‐in‐Chief of the Journal of Neurology, Associate Editor of Human Brain Mapping, Neurological Sciences, and Radiology; received compensation for consulting services from Alexion, Almirall, Biogen, Merck, Novartis, Roche, Sanofi; speaking activities from Bayer, Biogen, Celgene, Chiesi Italia SpA, Eli Lilly, Genzyme, Janssen, Merck‐Serono, Neopharmed Gentili, Novartis, Novo Nordisk, Roche, Sanofi, Takeda, and TEVA; participation in Advisory Boards for Alexion, Biogen, Bristol‐Myers Squibb, Merck, Novartis, Roche, Sanofi, Sanofi‐Aventis, Sanofi‐Genzyme, Takeda; scientific direction of educational events for Biogen, Merck, Roche, Celgene, Bristol‐Myers Squibb, Lilly, Novartis, Sanofi‐Genzyme; he receives research support from Biogen Idec, Merck‐Serono, Novartis, Roche, Italian Ministry of Health, Fondazione Italiana Sclerosi Multipla, and ARiSLA (Fondazione Italiana di Ricerca per la SLA). The University Hospital Basel (USB), as the employer of C.G., has received the following fees which were used exclusively for research support: (i) advisory board and consultancy fees from Actelion, Genzyme‐Sanofi, Novartis, GeNeuro, and Roche; (ii) speaker fees from Genzyme‐Sanofi, Novartis, GeNeuro and Roche; and (iii) research support from Siemens, GeNeuro, and Roche. C.G. is supported by the Swiss National Science Foundation (SNSF) grant PP00P3_176984, the Stiftung zur Förderung der gastroenterologischen und allgemeinen klinischen Forschung, and the EUROSTAR E!113,682 HORIZON2020. E.A.H. received honoraria for lecturing and advisory board activity from Biogen, Merck, and Sanofi‐Genzyme, unrestricted research grant from Merck, and is supported by grants from The Research Council of Norway (NFR, grant number 240102) and the South‐Eastern Health Authorities of Norway (grant number 257955). S.L. received compensation for consulting services and speaker honoraria from Biogen Idec, Novartis, TEVA, Genzyme, Sanofi, and Merck. S.M. received honoraria for lecturing and advisory board activity from UCB and Biogen and travel grant from Roche and Merck. M.M. has received research grants from the ECTRIMS‐MAGNIMS, the UK MS Society, and Merck and honoraria from Biogen, BMS Celgene, Ipsen, Merck, Novartis, and Roche. J.P. has received support for scientific meetings and honorariums for advisory work From Merck Serono, Novartis, Chugai, Alexion, Roche, Medimmune, Argenx, UCB, Mitsubishi, Amplo, Janssen, and Sanofi. Grants from Alexion, Roche, Medimmune, UCB, Amplo biotechnology. Patent ref. P37347WO and licence agreement Numares multimarker MS diagnostics Shares in AstraZenica. Acknowledges partial funding by highly specialized services NHS England. M.P. discloses travel/meeting expenses from Novartis, Janssen, Roche, and Merck, speaking honoraria from HEALTH&LIFE S.r.l., honoraria for consulting services from Biogen and research grants from Baroni Foundation. D.P. has received funding for travel from Merck, Genzyme/Sanofi‐Aventis, and Biogen, as well as speaking honoraria from Biogen, Novartis, and Merck. M.A.R. received consulting fees from Biogen, Bristol Myers Squibb, Eli Lilly, and Janssen, Roche and speaker honoraria from Bayer, Biogen, Bristol Myers Squibb, Bromatech, Celgene, Genzyme, Merck Healthcare Germany, Merck Serono SpA, Novartis, Roche, and Teva. She receives research support from the MS Society of Canada and Fondazione Italiana Sclerosi Multipla. She is Associate Editor for multiple sclerosis and related disorders. A.T. has been supported by grants from MRC (MR/S026088/1), NIHR BRC (541/CAP/OC/818837), and RoseTrees Trust (A1332 and PGL21/10079), has had meeting expenses from Merck, Biomedia and Biogen Idec, and was UK PI for two clinical trials sponsored by MEDDAY (MS‐ON—NCT02220244 and MS‐SPI2—NCT02220244). P.V. received speaker honoraria from Biogen Idec. O.C. is an NIHR Research Professor (RP‐2017‐08‐ST2‐004); acts as a consultant for Biogen, Merck, Novartis, Roche, and Teva; and has received research grant support from the MS Society of Great Britain and Northern Ireland, the NIHR UCLH Biomedical Research Centre, the Rosetree Trust, the National MS Society, and the NIHR‐HTA. M.M.S. serves on the editorial board of Neurology and Frontiers in Neurology, receives research support from the Dutch MS Research Foundation, Eurostars‐EUREKA, ARSEP, Amsterdam Neuroscience, MAGNIMS and ZonMW and has served as a consultant for or received research support from Atara Biotherapeutics, Biogen, Celgene/Bristol Meyers Squibb, Genzyme, MedDay and Merck. F.B.: Steering committee and iDMC member for Biogen, Merck, Roche, EISAI. Consultant for Roche, Biogen, Merck, IXICO, Jansen Combinostics. Research agreements with Novartis, Merck, Biogen, GE, Roche. Co‐founder and shareholder of Queen Square Analytics LTD. The remaining authors report no competing interests.

## Supporting information


**Data S1** Supporting Information

## Data Availability

Data from patients are controlled by the respective centers (listed in Supplementary Table 1) and therefore are not publicly available. Derived data supporting the findings of this study can be shared upon reasonable request by qualified investigators from the corresponding author, provided that the MAGNIMS consortium is acknowledged in any resulting publication.

## Appendix A

### A.1

Authors are members of the MAGNIMS network (magnetic resonance imaging in multiple sclerosis; https://www.magnims.eu/), which is a group of European clinicians and scientists with an interest in undertaking collaborative studies using MRI methods in multiple sclerosis, independent of any other organization. The group is run by a steering committee whose members are: Frederik Barkhof (Amsterdam), Nicola de Stefano (Siena), Jaume Sastre‐Garriga (Barcelona Co‐Chair), Olga Ciccarelli (London), Christian Enzinger (Graz), Massimo Filippi (Milan), Claudio Gasperini (Rome), Ludwig Kappos (Basel), Jacqueline Palace (Oxford), Menno Schoonheim (Amsterdam), Alex Rovira (Barcelona), Maria Assunta Rocca (Milan Co‐Chair), and Tarek Yousry (London).
