# Supplementary material for: More Than the Sum of Its Parts: Disrupted Core Periphery of Multiplex Brain Networks in Multiple Sclerosis
Source: Hum Brain Mapp. 2024 Dec 30;46(1):e70107. doi: 10.1002/hbm.70107 (PMC11685378; doi:10.1002/hbm.70107)
Supplement: Supplementary file 1 — Data S1 Supporting Information [file HBM-46-e70107-s001.docx]

**More than the sum of its parts: disrupted core-periphery of multiplex brain networks in multiple sclerosis**

# Supplementary Results

## Sensitivity analysis using the Brainnetome Atlas

When using the Brainnetome atlas to obtain a more fine-grained parcellation of the cortex (210 cortical and 14 subcortical regions), the multiplex core-periphery organization in the HC group followed closely the one observed with the Schaefer atlas (Supplementary Figure 3), with the multiplex core including on average subcortical GM structures and both sensorimotor and associative cortical areas. PwMS showed widespread deviations in regional coreness compared to the HC group, with the greatest effects observed at the level of deep GM structures (reduced coreness) (Supplementary Figure 4A). The anatomical distribution of the observed changes was such that topologically central nodes were generally more impacted than peripheral ones (which tended to have preserved or even increased coreness values), as expressed by the average κ = -0.11 (Hedges’ g = 0.63 *p* < 0.001) (Supplementary Figure 4B).

There was a significant effect of clinical phenotype on the weakening of the core-periphery structure of multimodal brain networks (F[3, 1044] = 7.50, *p* < 0.001). We observed, on average, progressively greater disruption in relapse-onset forms going from clinically-isolated syndrome (CIS) to participants with secondary-progressive multiple sclerosis (SPMS), and intermediate κ values in patients with primary-progressive (PPMS) forms (Supplementary Figure 4C). Patients with a lower physical disability (as measured through the Expanded Disability Status Scale, EDSS) had a stronger/less disrupted core-periphery organization (Hedges’ g= 0.32, *p* < 0.001), as well as patients with preserved cognition (measured with the Symbol Digit Modalities Test, SDMT) (Hedges’ g = 0.38, *p* < 0.001) (Supplementary Figure 4D-E).

## Sensitivity analysis on participants with non-atrophic brains

When restricting the analyses to participants with relatively preserved global brain volumes (age- and sex-adjusted BPF z-score based on the distribution in HC > -1.5; n = 1104: 765 CIS/MS, 339 HC), the results followed closely the ones of the main analyses in terms of deviation in regional coreness (Supplementary Figure 5A), coreness disruption index (average κ = -0.15, Hedges’ g = 0.54, *p* < 0.001; Supplementary Figure 5B), effect of phenotype (F[3, 761] = 3.90, *p* = 0.009; Supplementary Figure 5C), and association with levels of EDSS (Hedges’ g = 0.16, *p* = 0.04; Supplementary Figure 5D) and SDMT (Hedges’ g = 0.30, *p* < 0.001; Supplementary Figure 5E).

## Sensitivity analysis on single-site, non-harmonized, matrices

When conducting the analyses on non-harmonized matrices from the largest cohort (N = 420: 325 PwMS, 95 HC), similar effects were observed in terms of deviation in regional coreness (Supplementary Figure 6A) and coreness disruption index (average κ = -0.16, Hedges’ g = 0.40, *p* < 0.001; Supplementary Figure 6B). As for the effect of clinical phenotype, this was not significant (F[2, 322] = 1.323, *p* = 0.3; Supplementary Figure 6C), which however may be due to the reduced statistical power associated with the smaller sample size, with some clinical phenotypes only poorly represented (CIS = 0, RRMS = 238, SPMS = 51, PPMS = 36). Regarding the association with the levels of EDSS and SDMT, there was a trend towards higher κ values in patients with a lower physical disability (Hedges’ g = 0.21, *p* = 0.06; Supplementary Figure 6D), with stronger/less disrupted core-periphery organization in patients with preserved cognition (Hedges’ g = 0.27, *p* = 0.02; Supplementary Figure 6E).


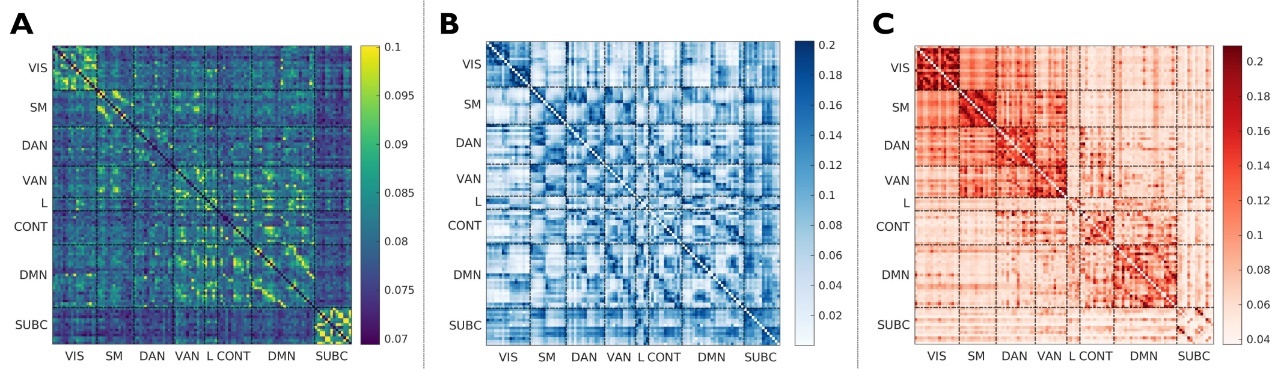


**Supplementary** Figure 1. **Average connectivity matrices in the healthy controls group.** Matrices of (**A**) morphological covariance, (**B**) structural connectivity, and (**C**) functional connectivity averaged over all healthy subjects. Matrices are ordered according to 7 canonical resting-state networks,^1^ plus a network of subcortical gray matter regions.

VIS = visual network; SM = somatomotor network; DAN = dorsal attention network; VAN = ventral attention network; L = limbic network; CONT = control network; DMN = default mode network; SUBC = subcortical network.


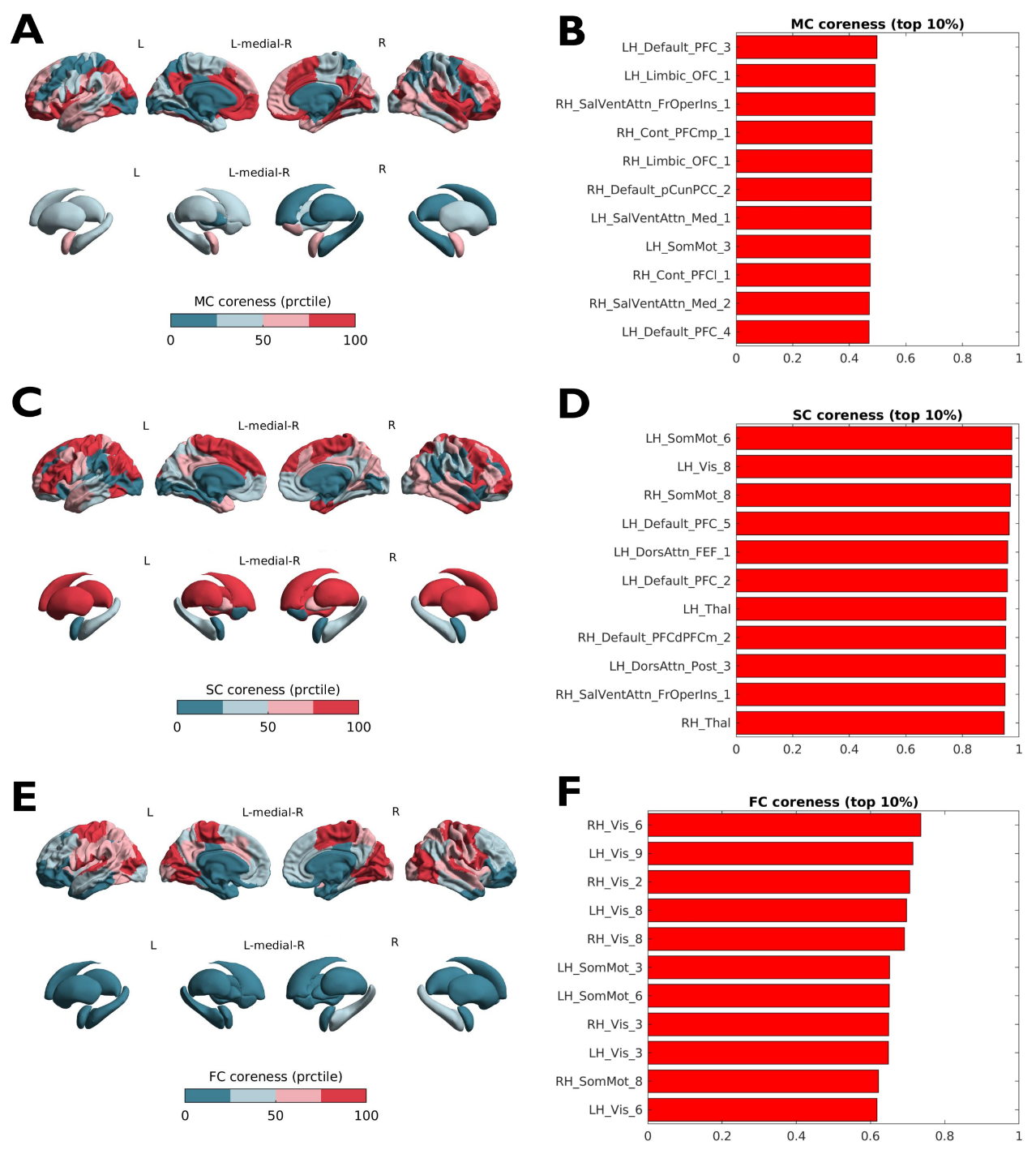


**Supplementary Figure 2. Average single-layer coreness in the healthy controls group.** Color-coded (*teal* to *red*) maps of coreness percentile ranks in the (**A**) morphological covariance, (**C**) structural connectivity, and (**E**) functional connectivity domains, superimposed on surface renderings of the cortex and subcortical structures. Images were obtained with the ENIGMA toolbox.^2^ (**B-D-F**) Highest 10% coreness nodes and corresponding absolute values are shown for the different layers. Nomenclature of cortical areas follows the 7-network Schaefer-100 parcellation.^1^

**
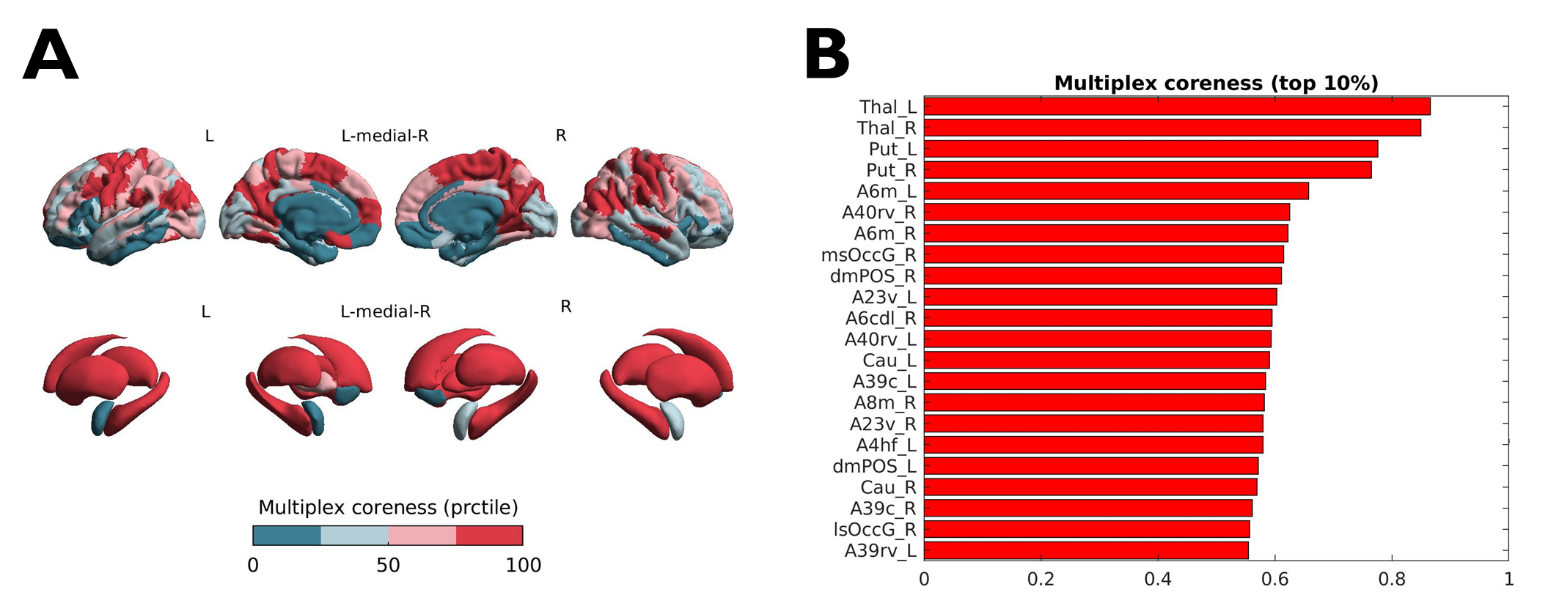
**

**Supplementary Figure 3. Average multiplex coreness in the healthy controls group when parcellating the cortex according to the Brainnetome atlas.** (**A**) Color-coded (*teal* to *red*) map of multiplex coreness percentile ranks superimposed on surface renderings of the cortex and subcortical structures. Image was obtained with the ENIGMA toolbox.^2^ (**B**) Highest 10% multiplex coreness nodes and corresponding absolute values are shown. Nomenclature of cortical areas is based on cytoarchitectonics according to the Brainnetome atlas.^3^ Macroanatomical labelling and assignment to the 7 canonical resting-state networks (in parentheses) is also provided in the legend below.

A6m = Superior Frontal Gyrus (Somatomotor Network); A40rv = Inferior Parietal Lobule (Somatomotor Network); msOccG = Lateral Occipital Cortex (Visual Network); dmPOS = Precuneus (Visual Network); A23v = (Visual / Default Mode Network); A6cdl = Precentral Gyrus (Dorsal Attention Network); A39c = Inferior Parietal Lobule (Visual Network); A8m = Superior Frontal Gyrus (Ventral Attention Network); A4hf = Precentral Gyrus (Somatomotor Network); A39c = Inferior Parietal Lobule (Visual Network); lsOccG = Lateral Occipital Cortex (Visual Network); A39rv = Inferior Parietal Lobule (Dorsal Attention Network).

**
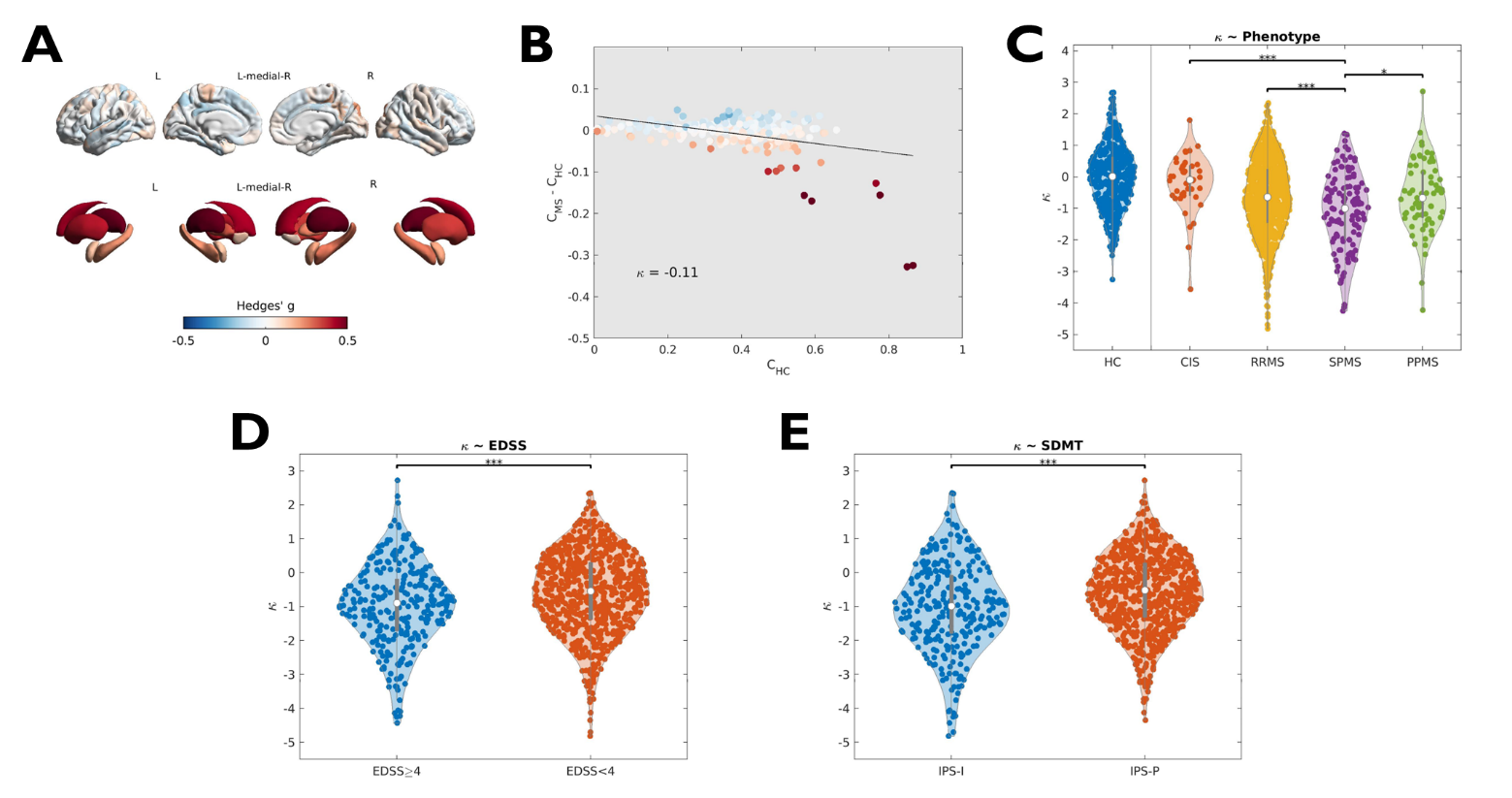
**

**Supplementary Figure 4. Results of the sensitivity analysis using the Brainnetome atlas.** (**A**) Color-coded (*blue* to *red*) map of effect sizes (Hedges’ g) of the between-group difference superimposed on surface renderings of the cortex and subcortical structures. Image was obtained with the ENIGMA toolbox.^2^ (**B**) Scatterplot showing, region-wise, the between-group difference in average regional coreness as a function of the average coreness in the HC group. The slope of the linear regression line corresponds to the coreness disruption index κ = -0.11. Each circle represents a brain region, color-coded as in panel A. (**C**) Violin plots showing the distributions of standardized κ values across different phenotypes. The distribution in healthy controls is also shown for reference. (*) Adjusted *p* < 0.05; (***) Adjusted *p* < 0.001. In (**D**) and (**E**), violin plots showing the distribution of κ values in patients with different levels of physical disability and cognitive performance, respectively. In (**C-E**), κ values are expressed as confounder-adjusted z-scores.


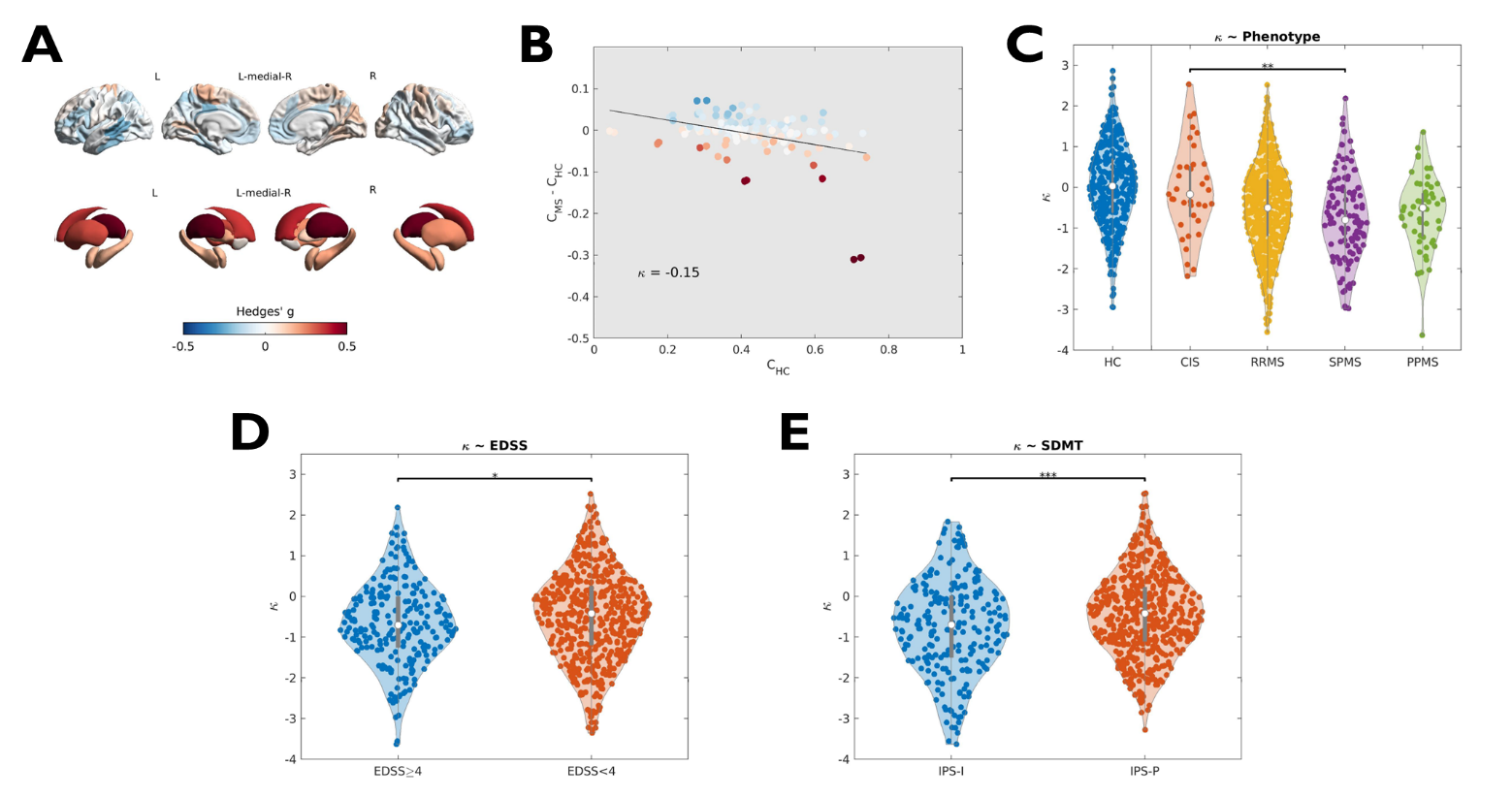


**Supplementary Figure 5. Results of the sensitivity analysis on participants with non-atrophic brains.** (**A**) Color-coded (blue to red) map of effect sizes (Hedges’ g) of the between-group difference superimposed on surface renderings of the cortex and subcortical structures. Image was obtained with the ENIGMA toolbox.^2^ (**B**) Scatterplot showing, region-wise, the between-group difference in average regional coreness as a function of the average coreness in the HC group. The slope of the linear regression line corresponds to the coreness disruption index κ = -0.15. Each circle represents a brain region, color-coded as in panel A. (**C**) Violin plots showing the distributions of standardized κ values across different phenotypes. The distribution in healthy controls is also shown for reference. (*) Adjusted *p* < 0.05; (***) Adjusted *p* < 0.001. In (**D**) and (**E**), violin plots showing the distribution of κ values in patients with different levels of physical disability and cognitive performance, respectively. In (**C-E**), κ values are expressed as confounder-adjusted z-scores.

**
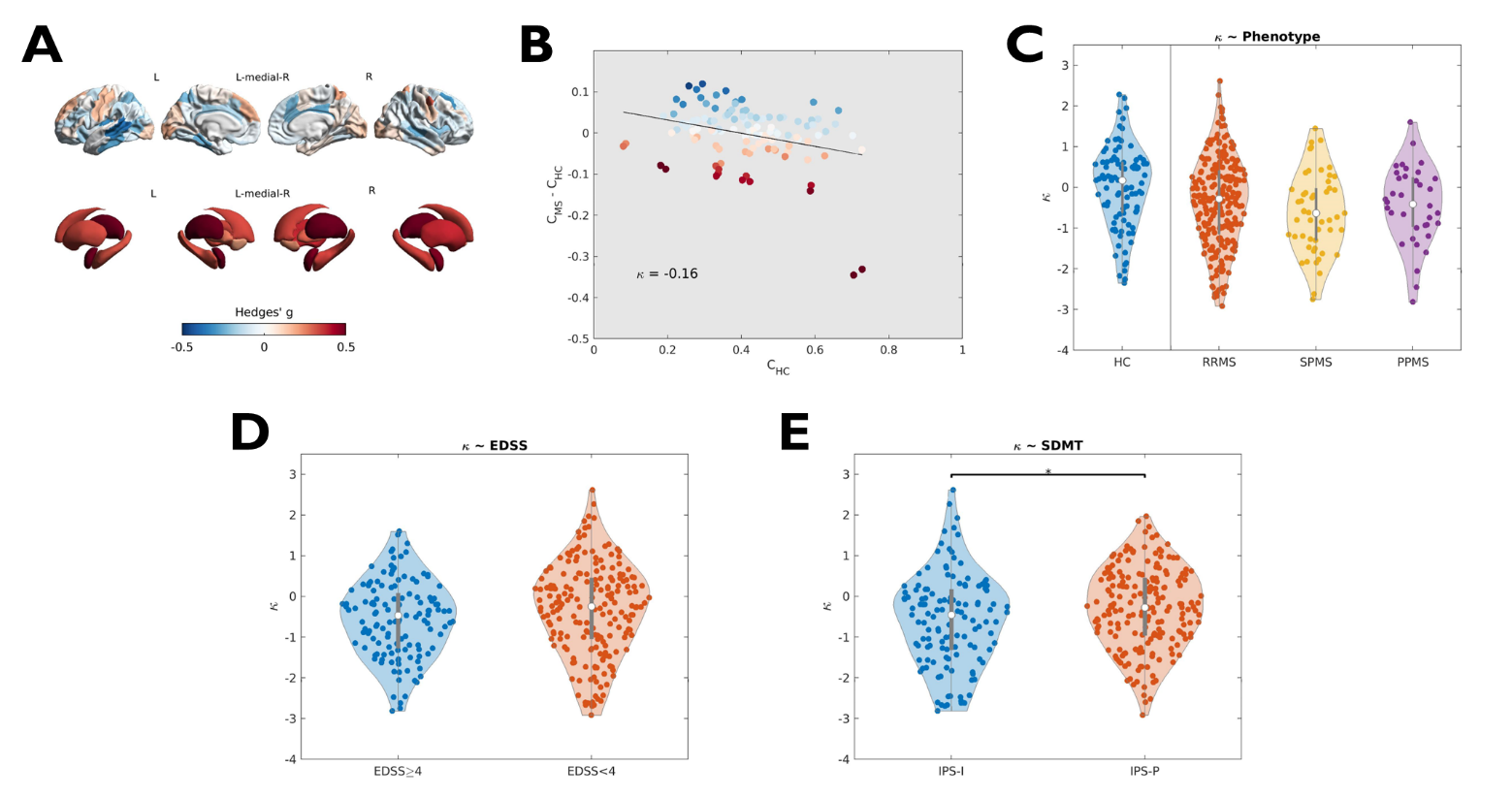
**

**Supplementary Figure 6. Results of the sensitivity analysis on single-site, non-harmonized, matrices.** (**A**) Color-coded (blue to red) map of effect sizes (Hedges’ g) of the between-group difference superimposed on surface renderings of the cortex and subcortical structures. Image was obtained with the ENIGMA toolbox.^2^ (**B**) Scatterplot showing, region-wise, the between-group difference in average regional coreness as a function of the average coreness in the HC group. The slope of the linear regression line corresponds to the coreness disruption index κ = -0.15. Each circle represents a brain region, color-coded as in panel A. (**C**) Violin plots showing the distributions of standardized κ values across different phenotypes. The distribution in healthy controls is also shown for reference. In (**D**) and (**E**), violin plots showing the distribution of κ values in patients with different levels of physical disability and cognitive performance, respectively. (*) Adjusted *p* < 0.05. In (**C-E**), κ values are expressed as confounder-adjusted z-scores.


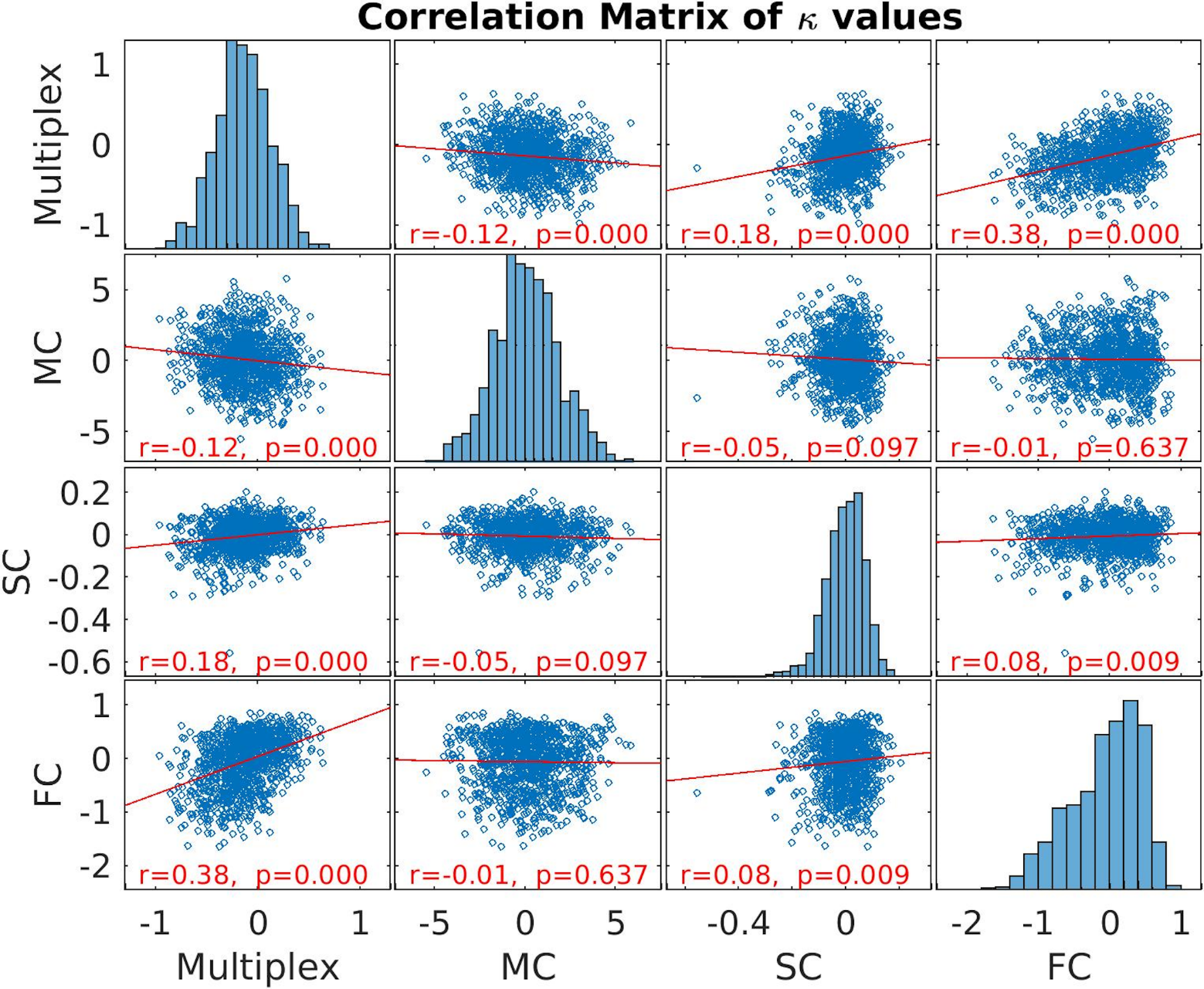


**Supplementary Figure 7. Correlations between coreness disruption in the multiplex and single-layer domains.** Correlogram showing, in PwMS, the correlations (scatterplots and Pearson correlation coefficient) between κ values in the multiplex and single-layer domains, along with their distributions (on the diagonal). Disruption of the multiplex core-periphery organization was mainly associated with homologous phenomena in the structural (SC) and functional (FC) layers, with a slight opposite contribution of the morphological covariance (MC) layer.

| **Centre** | Amsterdam | | | Barcelona | | | Basel | | | Graz | | |
| --- | --- | --- | --- | --- | --- | --- | --- | --- | --- | --- | --- | --- |
| **Vendor, Model** | GE, Signa | | | Siemens, Trio | | | Siemens, Prima | | | Siemens, Prisma | | |
| **Years of recruitment** | 2008-2012 | | | 2016-2019 | | | 2019-2021 | | | 2021-2022 | | |
| **Modality** | **3D T1w** | **dMRI** | **rs-fMRI** | **3D T1w** | **dMRI** | **rs-fMRI** | **3D T1w** | **dMRI** | **rs-fMRI** | **3D T1w** | **dMRI** | **rs-fMRI** |
| **Voxel dimensions (mm)** | 0.9x0.9x1 | 2x2x2.4 | 3.3x3.3x3 | 0.94x0.94x0.94 | 1.5x1.5x1.51 | 3x3x3 | 1x1x1 | 1.8x1.8x1.8 | 2x2x2 | 1x1x1 | 1.5x1.5x1.5 | 2x2x2 |
| **TR (ms)** | 7.8 | 13000 | 2200 | 1800 | 14800 | 2000 | 5000 | 4500 | 768 | 1900 | 3318 | 1000 |
| **TE (ms)** | 3 | 91 | 35 | 3 | 103 | 19 | 3 | 75 | 37 | 2.7 | 87.4 | 35 |
| **TI (ms)** | 450 | - | - | 900 | - | - | - | - | - | 900 | - | -- |
| **FA (°)** | 12 | 90 | 80 | 9 | 90 | 90 |  | 90 | 52 | 9 | 78 | 70 |
| **Slices, Orientation** | 206, sagittal | 53, axial | 40, axial | 240, sagittal | 100, axial | 40, axial | 176, sagittal | 80, axial | 72, axial | 176, sagittal | 96, axial | 54, axial |
| **Directions/Time points** | - | 30 | 202 | - | 60 | 450 | - | 137 | 457 | - | 96 | 300 |
| **b-values (s/mm^2^)** | - | 1000 | - | - | 1000 | - | - | 700, 1000, 2000, 3000 | - | - | 800, 1600, 2500 | - |
| **EPI distortion correction** | - | Fieldmap-less | Fieldmap-less | - | Phase-difference | Fieldmap-less | - | PEPOLAR | PEPOLAR | - | PEPOLAR | Fieldmap-less |
|  | **Number of participants** | | | **Number of participants** | | | **Number of participants** | | | **Number of participants** | | |
| **HC** | 95 | | | 8 | | | 15 | | | 50 | | |
| **CIS** | - | | | - | | | - | | | 3 | | |
| **RRMS** | 238 | | | 51 | | | 8 | | | 127 | | |
| **SPMS** | 51 | | | 9 | | | 3 | | | 10 | | |
| **PPMS** | 36 | | | - | | | 3 | | | 3 | | |
| **QC exclusions** | 8 | | | 1 | | | 1 | | | 1 | | |

| **Centre** | London | | | Mainz | | | Milan | | | Naples I | | |
| --- | --- | --- | --- | --- | --- | --- | --- | --- | --- | --- | --- | --- |
| **Vendor, Model** | Philips, Achieva | | | Siemens, Trio | | | Philips, Ingenia | | | Siemens, Trio | | |
| **Years of recruitment** | 2019-2021 | | | 2017-2019 | | | 2017-2020 | | | 2016-2018 | | |
| **Modality** | **3D T1w** | **dMRI** | **rs-fMRI** | **3D T1w** | **dMRI** | **rs-fMRI** | **3D T1w** | **dMRI** | **rs-fMRI** | **3D T1w** | **dMRI** | **rs-fMRI** |
| **Voxel dimensions (mm)** | 1x1x1 | 2x2x2 | 3x3x3 (0.5-mm gap) | 1x1x1 | 2.5x2.5x2.5 | 3x3x2 (1-mm gap) | 1x1x1 | 2.3x2.3x2.3 | 2.5x2.5x3 | 0.8x0.8x0.8 | 2.2x2.2x2.2 | 3x3x4 (1-mm gap) |
| **TR (ms)** | 7 | 6079 | 4000 | 1900 | 9000 | 3060 | 7 | 5900 | 1560 | 3000 | 7400 | 2500 |
| **TE (ms)** | 3.2 | 96 | 25 | 2.5 | 102 | 30 | 3.2 | 78 | 35 | 2.4 | 88 | 50 |
| **TI (ms)** | - | - | - | 900 | - | - | 1000 | - | - | 1000 | - | - |
| **FA (°)** | 8 | 90 | 90 | 9 | 90 | 90 | 8 | 90 | 70 | 9 | 90 | 90 |
| **Slices, Orientation** | 176, sagittal | 72, axial | 43, axial | 192, sagittal | 62, axial | 49, axial | 204, sagittal | 56, axial | 48, axial | 224, sagittal | 60, axial | 30, axial |
| **Directions/Time points** | - | 76 | 100 | - | 30 | 205 | - | 96 | 320 | - | 64 | 200 |
| **b-values (s/mm^2^)** | - | 1000, 2000, 2800 | - | - | 900 | - | - | 700, 1000, 2850 | - | - | 1000 | - |
| **EPI distortion correction** | - | PEPOLAR | Fieldmap-less | - | Fieldmap-less | Fieldmap-less | - | PEPOLAR | Fieldmap-less | - | Fieldmap-less | Fieldmap-less |
|  | **Number of participants** | | | **Number of participants** | | | **Number of participants** | | | **Number of participants** | | |
| **HC** | 16 | | | 55 | | | 35 | | | 52 | | |
| **CIS** | 34 | | | - | | | - | | | - | | |
| **RRMS** | 25 | | | 50 | | | 29 | | | 30 | | |
| **SPMS** | - | | | - | | | 26 | | | 15 | | |
| **PPMS** | - | | | - | | | 7 | | | 7 | | |
| **QC exclusions** | - | | | 3 | | | 3 | | | 1 | | |

| **Centre** | Naples II | | | Oslo | | | Oxford | | | Siena | | | Verona | | |
| --- | --- | --- | --- | --- | --- | --- | --- | --- | --- | --- | --- | --- | --- | --- | --- |
| **Vendor, Model** | GE, Discovery | | | GE, Discovery | | | Siemens, Prisma | | | Philips, Achieva | | | Philips, Achieva | | |
| **Years of recruitment** | 2019-2022 | | | 2016-2019 | | | 2018-2019 | | | 2017-2021 | | | 2015-2017 | | |
| **Modality** | **3D T1w** | **dMRI** | **rs-fMRI** | **3D T1w** | **dMRI** | **rs-fMRI** | **3D T1w** | **dMRI** | **rs-fMRI** | **3D T1w** | **dMRI** | **rs-fMRI** | **3D T1w** | **dMRI** | **rs-fMRI** |
| **Voxel dimensions (mm)** | 1x1x1 | 2x2x2 | 2.3x2.3x3 | 1x1x1 | 2x2x2 | 3x3x3 (1-mm gap) | 1x1x1 | 2x2x2 | 2.4x2.4x2.4 | 1x1x1 | 2.5x2.5x2.5 | 1.9x1.9x4 | 1x1x1 | 2x2x2 | 1.8x1.8x4 |
| **TR (ms)** | 7.0 | 7302 | 1500 | 8.2 | 8150 | 2250 | 2040 | 3600 | 735 | 10 | 7053.1 | 3000 | 8.2 | 9300 | 2600 |
| **TE (ms)** | 3.0 | 79.2 | 19 | 3.2 | 83.1 | 30 | 4.7 | 92 | 39 | 4 | 96.4 | 35 | 3.8 | 109 | 35 |
| **TI (ms)** | 650 | - | - | 450 | - | - | 900 | - | - | - | - | - | - | - | - |
| **FA (°)** | 9 | 90 | 90 | 12 | 90 | 79 | 8 | 78 | 52 | 8 | 90 | 90 | 8 | 90 | 90 |
| **Slices, Orientation** | 206, sagittal | 66, axial | 44, axial | 188, sagittal | 67, axial | 43, axial | 192, sagittal | 72, axial | 64, axial | 256, sagittal | 50, axial | 30, axial | 180, sagittal | 62, axial | 35, axial |
| **Directions/Time points** | - | 64 | 320 | - | 60 | 200 | - | 100 | 490 | - | 32 | 200 | - | 96 | 225 |
| **b-values (s/mm^2^)** | - | 2000 | - | - | 1000 | - | - | 1000, 2000 | - | - | 900 | - | - | 700, 2000 | - |
| **EPI distortion correction** | - | PEPOLAR | Fieldmap-less | - | PEPOLAR | Phase-difference | - | PEPOLAR | Fieldmap-less | - | Fieldmap-less | Fieldmap-less | - | PEPOLAR | Fieldmap-less |
|  | **Number of participants** | | | **Number of participants** | | | **Number of participants** | | | **Number of participants** | | | **Number of participants** | | |
| **HC** | 18 | | | 24 | | | 17 | | | 30 | | | 21 | | |
| **CIS** | 1 | | | - | | | - | | | - | | | 3 | | |
| **RRMS** | 51 | | | 56 | | | 16 | | | 92 | | | 44 | | |
| **SPMS** | 5 | | | 1 | | | - | | | - | | | 1 | | |
| **PPMS** | 5 | | | 1 | | | - | | | 6 | | | 1 | | |
| **QC exclusions** | 5 | | | 1 | | | 3 | | | 2 | | | 4 | | |

dMRI = diffusion MRI; rs-fMRI = resting-state functional MRI; TR = repetition time; TE = echo time; TI = inversion time; FA = flip angle; EPI = echo-planar imaging; PEPOLAR = Phase-encoding polarity; CIS = clinically isolated syndrome; HC = healthy controls; PPMS = primary-progressive multiple sclerosis; QC = quality control; RRMS = relapsing-remitting multiple sclerosis; SPMS = secondary-progressive multiple sclerosis; QC = quality control.

**Supplementary Table 1. MRI acquisition protocols.**

| **ROI name** | **Hedges’ g** | **FDR-adjusted *p*** |
| --- | --- | --- |
| LH_Thal | 0.89 | 0.004 |
| RH_Thal | 0.87 | 0.004 |
| LH_Cau | 0.41 | 0.004 |
| RH_Cau | 0.39 | 0.004 |
| LH_Put | 0.36 | 0.004 |
| RH_Put | 0.29 | 0.004 |
| RH_Pall | 0.22 | 0.007 |
| RH_Hipp | 0.20 | 0.008 |
| LH_Pall | 0.20 | 0.01 |
| LH_Cont_Cing_1 | -0.20 | 0.008 |
| RH_Amyg | 0.19 | 0.009 |
| LH_Hipp | 0.18 | 0.01 |
| LH_Default_Temp_2 | -0.18 | 0.01 |
| LH_Cont_Par_1 | -0.18 | 0.02 |
| LH_DorsAttn_Post_5 | -0.16 | 0.02 |
| LH_Default_Par_1 | -0.15 | 0.05 |
| RH_Default_PFCdPFCm_1 | -0.15 | 0.05 |

**Supplementary Table 2. Results of the between-group comparison in terms of multiplex regional coreness.** Regions for which a significant difference emerged at the PwMS vs HC comparison are shown, along with corresponding effect sizes (Hedges’ g) and FDR-adjusted *p* values. The nomenclature of cortical areas follows the 7-network Schaefer-100 parcellation.^1^

**References**

1. Schaefer A, Kong R, Gordon EM, et al. Local-Global Parcellation of the Human Cerebral Cortex from Intrinsic Functional Connectivity MRI. Cereb Cortex. 2018;28:3095–3114.

2. Larivière S, Paquola C, Park B, et al. The ENIGMA Toolbox: multiscale neural contextualization of multisite neuroimaging datasets. Nat Methods. Nature Publishing Group; 2021;18:698–700.

3. Fan L, Li H, Zhuo J, et al. The Human Brainnetome Atlas: A New Brain Atlas Based on Connectional Architecture. Cereb Cortex. 2016;26:3508–3526.
